# Phosphorus and Nitrogen Regulate Arbuscular Mycorrhizal Symbiosis in *Petunia hybrida*

**DOI:** 10.1371/journal.pone.0090841

**Published:** 2014-03-07

**Authors:** Eva Nouri, Florence Breuillin-Sessoms, Urs Feller, Didier Reinhardt

**Affiliations:** 1 Dept. of Biology, University of Fribourg, Fribourg, Switzerland; 2 Institute of Plant Science, University of Bern, Bern, Switzerland; Radboud University Medical Centre, NCMLS, Netherlands

## Abstract

Phosphorus and nitrogen are essential nutrient elements that are needed by plants in large amounts. The arbuscular mycorrhizal symbiosis between plants and soil fungi improves phosphorus and nitrogen acquisition under limiting conditions. On the other hand, these nutrients influence root colonization by mycorrhizal fungi and symbiotic functioning. This represents a feedback mechanism that allows plants to control the fungal symbiont depending on nutrient requirements and supply. Elevated phosphorus supply has previously been shown to exert strong inhibition of arbuscular mycorrhizal development. Here, we address to what extent inhibition by phosphorus is influenced by other nutritional pathways in the interaction between *Petunia hybrida* and *R. irregularis*. We show that phosphorus and nitrogen are the major nutritional determinants of the interaction. Interestingly, the symbiosis-promoting effect of nitrogen starvation dominantly overruled the suppressive effect of high phosphorus nutrition onto arbuscular mycorrhiza, suggesting that plants promote the symbiosis as long as they are limited by one of the two major nutrients. Our results also show that in a given pair of symbiotic partners (*Petunia hybrida* and *R. irregularis*), the entire range from mutually symbiotic to parasitic can be observed depending on the nutritional conditions. Taken together, these results reveal complex nutritional feedback mechanisms in the control of root colonization by arbuscular mycorrhizal fungi.

## Introduction

### Plant nutrition and AM symbiosis

In many environments, plant growth is limited by inadequate nutrient supply [1], [2]. This condition is alleviated by a mutualistic association with soil fungi of the order *Glomeromycota* which provide the plant host with diverse mineral nutrients in exchange for assimilates [3]. This symbiosis, referred to as arbuscular mycorrhiza (AM), emerged approximately 450 Ma ago, and is thought to have facilitated the colonization of land by early vascular plants [4]. The fungal mycelium emanating from the root system reaches far beyond the rhizosphere and therefore can acquire nutrients from soil volumes to which roots have no access [5]. Furthermore, fungal hyphae are much thinner than roots [6], allowing them to explore small cracks in the micrometer range that are inaccessible to roots. Besides better nutrient supply, mycorrhizal associations provide other benefits to plants, such as increased drought tolerance and disease resistance [7]–[9].

The predominant nutrient element acquired through AM is phosphorus (P), which is delivered to the plant in its inorganic oxidized form phosphate (P_i_) [10]. Furthermore, nitrogen (N) can be transferred to mycorrhizal plants through the fungal symbiont [11]–[14], involving a complex pathway that has been elucidated in considerable detail in recent years [15]–[17]. Apart from these central macronutrients, sulfur (S) [18], [19] and several micronutrients can be delivered to plants through AM fungi [20], [21].

Mycorrhizal plants have two routes of nutrient acquisition, the direct pathway (DP) through the root epidermis and its root hairs, (representing the only uptake route of non-mycorrhizal plants), and the mycorrhizal pathway (MP) through the fungal mycelium that delivers nutrients to the root cortex through the arbuscules. Surprisingly, P_i_ supply through the two pathways is not necessarily additive [22]. The contribution of the DP can be reduced in mycorrhizal plants, via the repression of constitutive phosphate transporter (PT) genes, to the extent that the plant becomes almost entirely dependent on the MP [23], [24]. Hence, total P nutrition of mycorrhizal plants is not necessarily higher than in non-mycorrhizal plants. This effect is one possible reason for the observation that mycorrhizal colonization does not always result in a net gain of plant growth, i.e. in a positive mycorrhizal growth response (MGR). Indeed, even negative MGRs occur quite frequently depending on the pairing of host and fungal symbiont [25], [26], raising the question why such interactions are tolerated by the plant. A plausible explanation for neutral or negative growth effects could be that AM confer a benefit other than growth promotion, e.g. a qualitative benefit, or that the benefit is not evident under the respective experimental conditions.

Modern agricultural practice has in many instances resulted in progressively reduced AM fungal diversity and frequency, an effect that is believed to be related to tillage methods [27] and to the use of mineral fertilizers [28], [29]. In particular, P_i_ has long been known to negatively impact on AM [30]–[38]. Inhibition of AM development by P_i_ is a systemic effect that depends on the nutritional status of the shoot. Since AM fungi can consume a considerable fraction of the assimilates of their hosts [26], [39], the suppression of AM by high P_i_ levels can be regarded as an energy-saving negative feedback mechanism under conditions under which the plant is optimally supplied with nutrients without the fungal symbiont. However, inhibition of AM development by P_i_ can potentially lead to starvation for other nutrients for which the DP is not sufficiently active, hence the P_i_ effect may reduce plant fitness depending on the supply with other nutrients. This raises the question whether the inhibition of AM development by P_i_ may become attenuated when plants are limited for other nutrients. Indeed, such nutritional interactions have been observed under natural conditions [40], however, these findings need to be substantiated under controlled conditions.

The aim of this study was to evaluate which nutrients, besides P, influence AM development, and with which nutritional pathway the P-pathway interacts. First, the major nutrient elements P, N, K, S, Mg, Ca, and Fe were tested for inhibitory effects on mycorrhizal colonization of *Petunia hybrida* by *Rhizophagus irregularis*. These experiments, together with previous findings [34], showed that strong nutritional repression of AM is specific to P. Secondly, to evaluate how the starvation for other nutrients influences the P-effect, combinatorial treatments of plants were performed with nutrient mixtures containing high P_i_ levels, but lacking several other nutrients alone or in combination. Our results show that the P- and N-related pathways interact, and that the AM-promoting effect of low N supply is dominant over the AM-suppressive effect of high P_i_ supply. These observations can serve as a basis to approach the underlying regulatory mechanisms with genetic means. Our results also show that in a given AM system (in this case *P. hybrida* and *R. irregularis*), nutrient supply has a strong influence not only on plant growth and nutrient content, but also on AM colonization and MGR. Depending on the nutritional context, AM can result in strong promotion of plant growth or in a reduction in qualitative and/or quantitative traits. Hence, the outcome of AM symbiosis is highly context-dependent.

## Materials and Methods

### Plant material, fungal inoculum and growth conditions

Two *Petunia hybrida* lines with indistinguishable interaction with arbuscular mycorrhizal fungi were used: Mitchell diploid (line W115) and line W138 (experiment 2; see below). Seeds were germinated on seedling substrate (Klasmann, http://www.klasmann-deilmann.com). After four weeks, plantlets were transferred to small pots (volume: 20 ml) of a sterilized mixture of 70% sand with 30% unfertilized soil (v/v; further referred to as “substrate”) for another two weeks. The substrate was sterilized by heating to 90°C for 3 h with vapor. Subsequently, plantlets were transferred to larger pots (volume: 150 ml) with the same substrate and inoculated with 1 teaspoon (*ca*. 10 g) of mycorrhizal inoculum per plant directly to the root system. Inoculum of *R. irregularis* (MUCL 43204) was produced in leek pot cultures. The inoculum consisted of a mixture of soil and roots, and was tested for the presence of spores before use. In general, the plants were cultured in growth chambers with a day:night cycle (16 h day at 25°C; 8 h night at 20°C) with a photosynthetic photon flux density (PPFD) of 250 µmol m^−2^ s^−1^ (Sylvania 36 W Luxline-Plus). Nutrient composition of the substrate (Table S1) was determined by extraction of 10 g substrate in 20 ml 0.5 M NaHCO_3_ (pH = 8.5) for 30 min under shaking. For the determination of sodium content, 10 g of substrate was extracted with 20 ml distilled water. Nutrient levels in the resulting soil extracts were determined as described below.

### Experimental design and nutrient treatments

**Figure 1:**
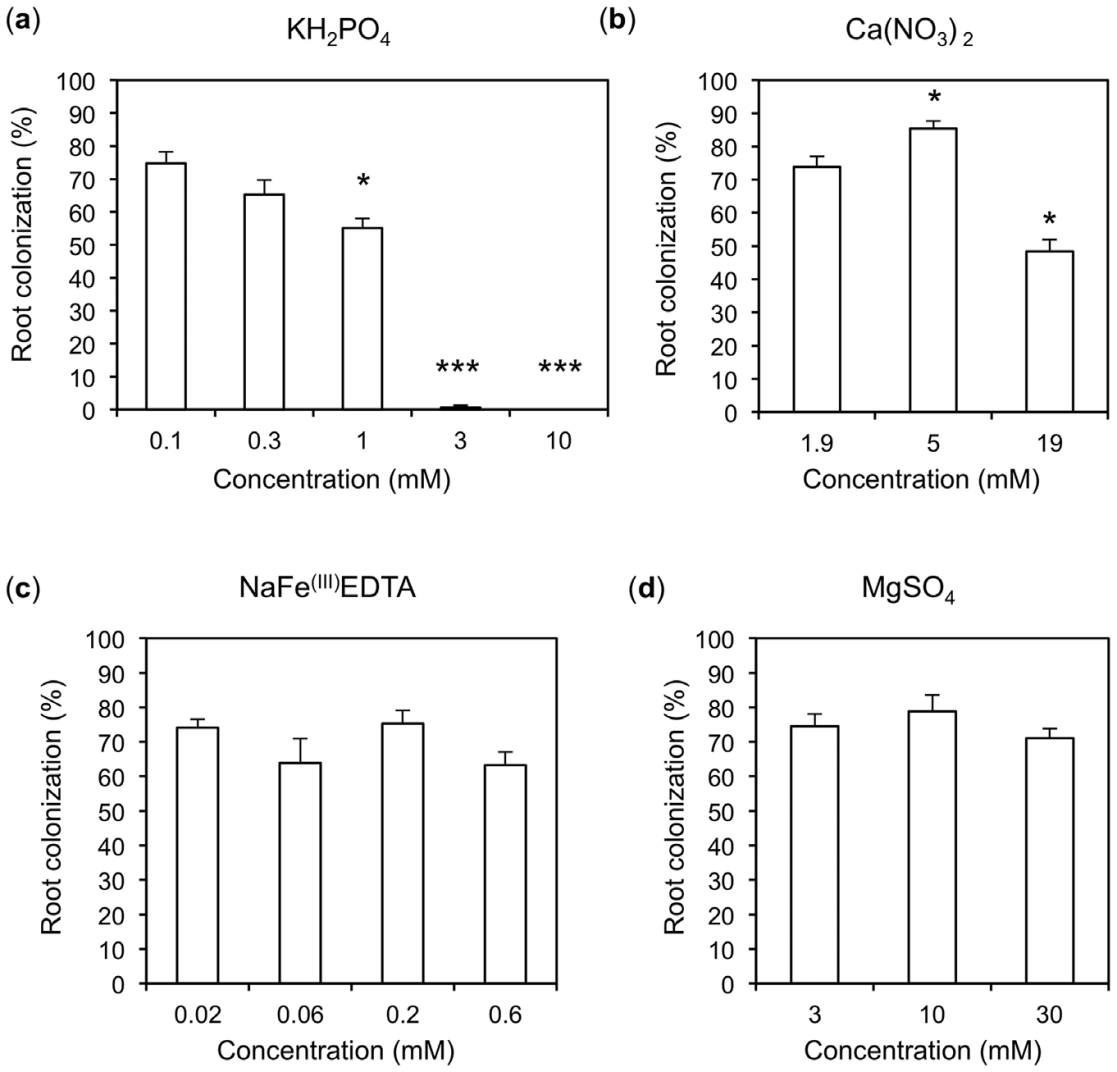
Exogenous phosphate and nitrate inhibit root colonization by *Rhizophagus irregularis*. Plants inoculated with *Rhizophagus irregularis* were watered with the basic nutrient solution, additionally supplemented with the indicated nutrient concentrations. The first column to the left in each graph corresponds to the concentration in the basic nutrient solution, except for KH_2_PO_4_ (see Table S2); the other columns represent elevated nutrient levels as indicated. Columns represent the average of four replicate plants with standard deviations.

**Figure 2:**
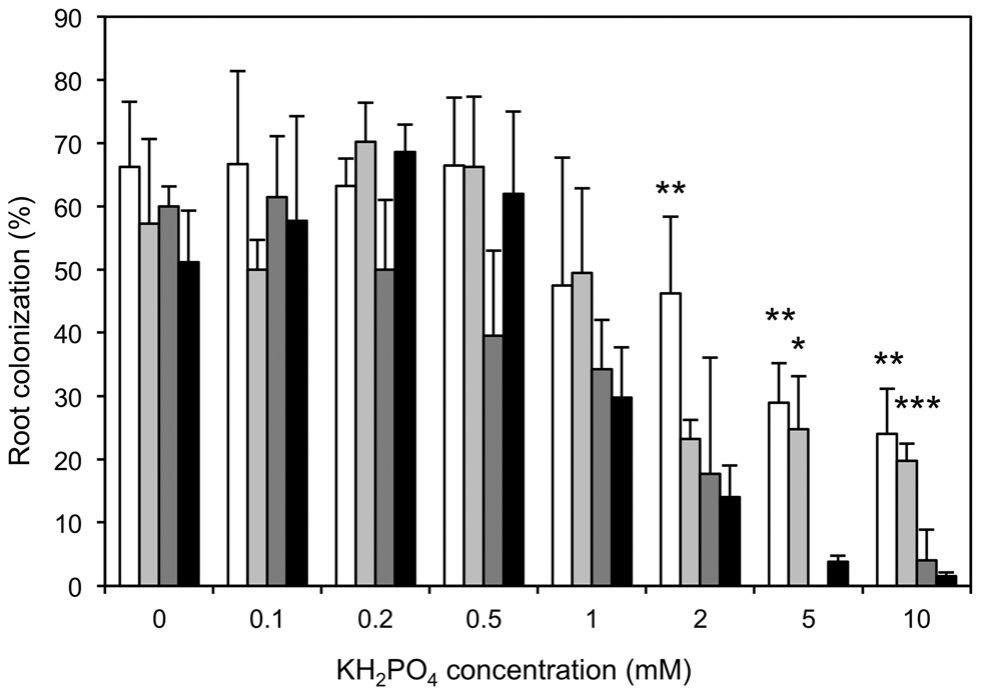
Inhibition of AM colonization by exogenous phosphate depends on the supply with other nutrients. KH_2_PO_4_ was applied to inoculated plants at the indicated concentrations either with basic nutrient solution (black columns) or alone (white columns). Additional treatments involved the application of KH_2_PO_4_ with only micronutrients (light grey) or only macronutrients (dark grey), respectively. Columns represent the average of four replicate plants with standard deviations. Asterisks indicate significant differences between phosphate alone (white bars) and phosphate with micronutrients (light grey bars), respectively, vs. the treatment with a combination of P_i_ and basic nutrient solution (black bars).

**Figure 3:**
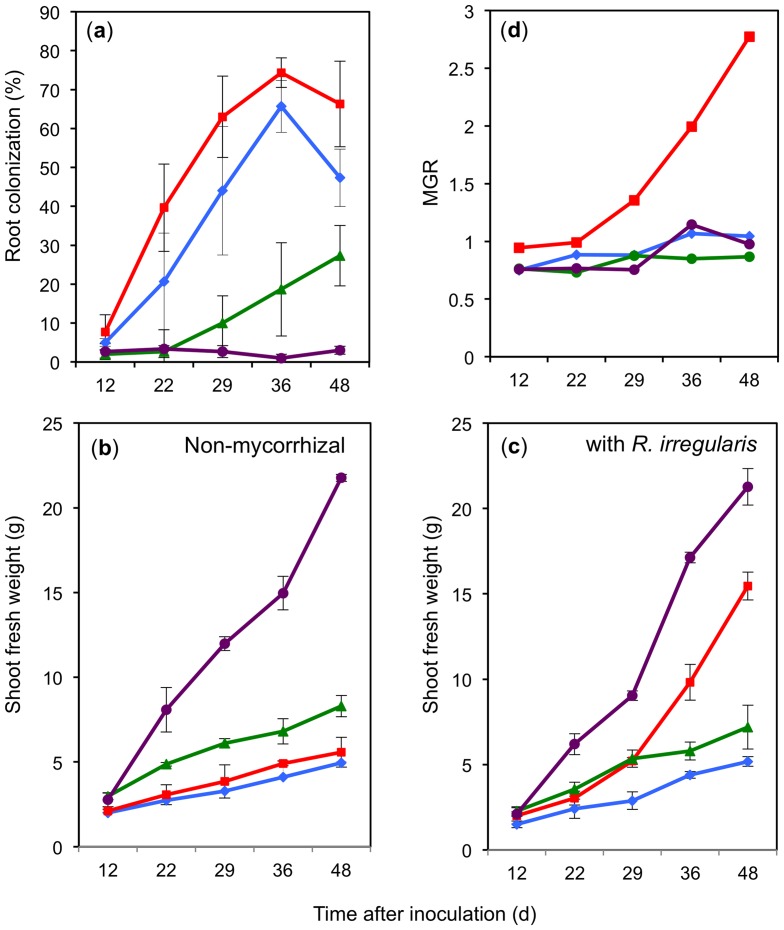
Dynamics of fungal root colonization and plant growth under different nutritional conditions. Plants inoculated or not with *R. irregularis* were supplied with water (blue), basic nutrient solution (red), 5 mM KH_2_PO_4_ (green), or with a combination of 5 mM KH_2_PO_4_ and basic nutrient solution (purple). Samples were harvested at the indicated time points to determine root colonization (a), shoot fresh weight (b,c), and mycorrhizal growth response (MGR; (d)). MGR is defined as the ratio of the shoot weight of mycorrhizal versus non-mycorrhizal control plants. Values are the mean of three biological replicates. Error bars represent standard deviations.

**Figure 4:**
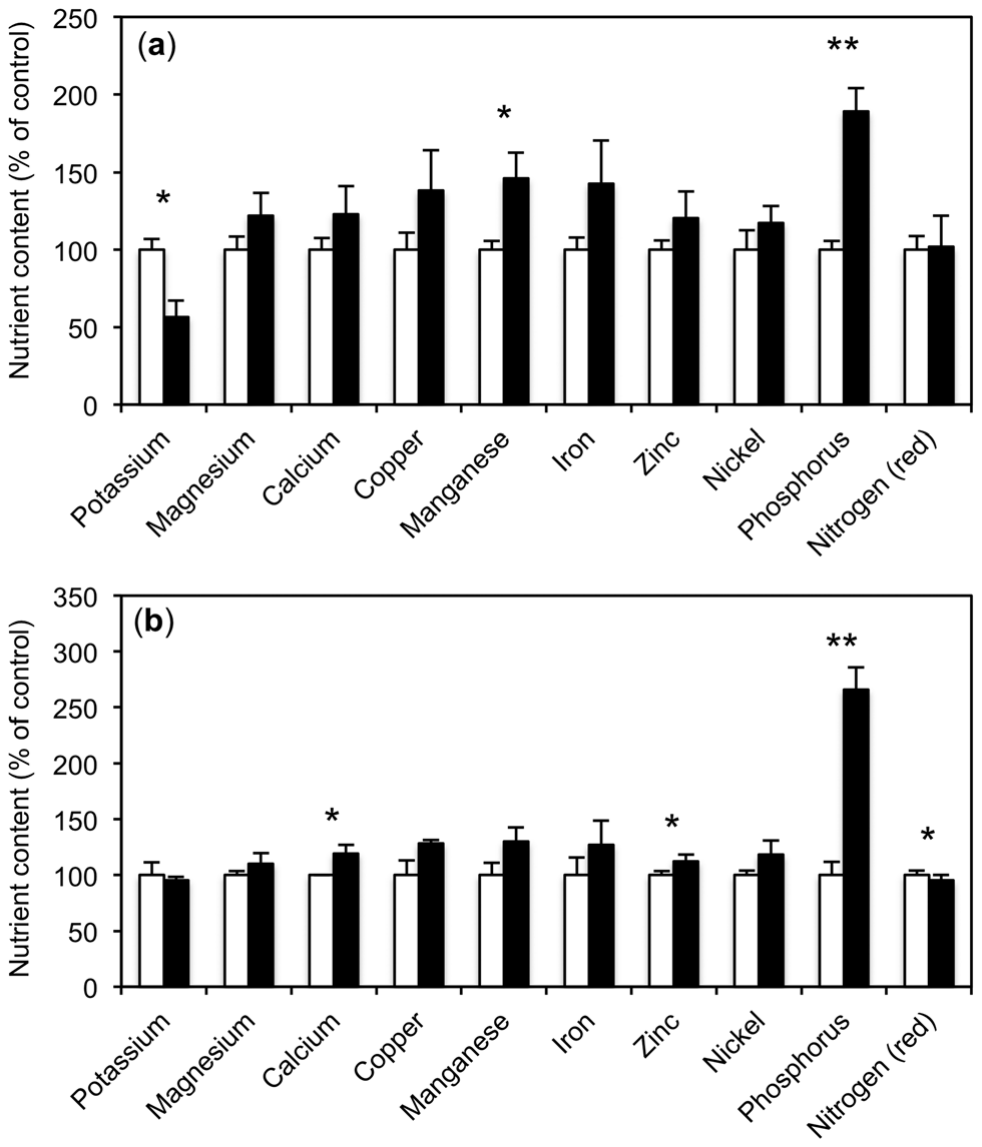
*R. irregularis* increases nutrient content of plants supplied with water or with nutrient solution. Nutrient levels in leaves were determined 36 days after inoculation in the plants shown in Figure 3. Values are the mean of three biological replicates. Error bars represent standard deviations. Asterisks indicate significant differences between mycorrhizal roots (black columns) and non-mycorrhizal controls (white columns). (**a**) Plants were fertilized with basic nutrient solution. Values are expressed relative to the non-mycorrhizal fertilized controls that were set to 100% for each nutrient. (**b**) As in (a), but without nutrient solution. Values are expressed relative to the non-colonized water-treated controls that were set to 100% for each nutrient.

**Figure 5:**
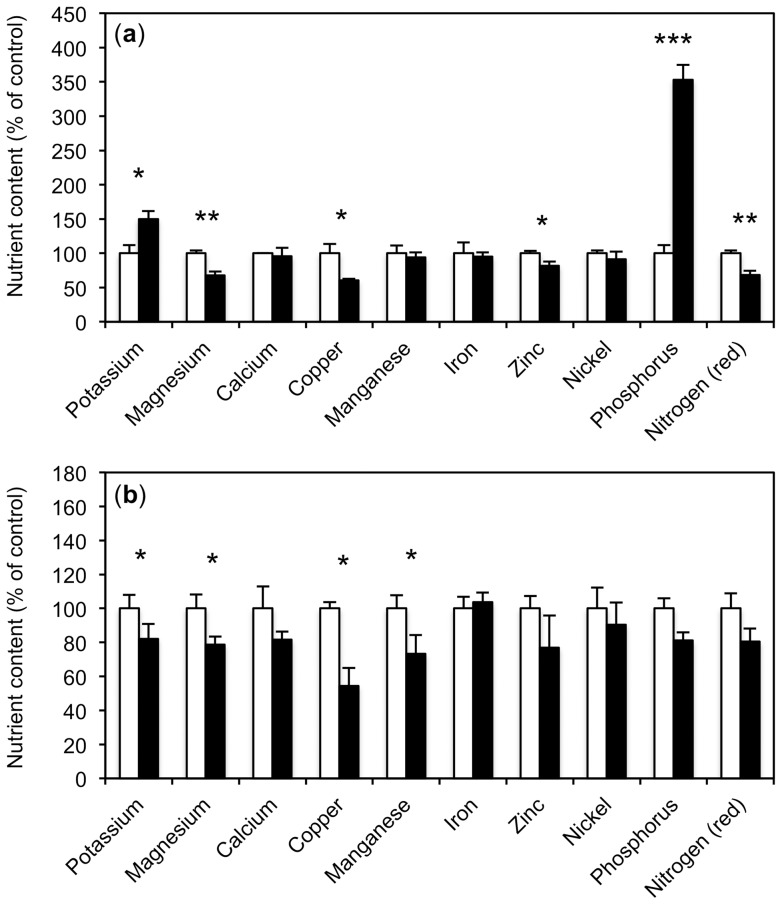
Treatment with KH_2_PO_4_ alone causes depletion of other nutrients and interferes with nutrient acquisition in mycorrhizal roots. Nutrient levels in leaves were determined 36 days after inoculation in the plants shown in Figure 3. Values are the mean of three replicates. Error bars represent standard deviations. Asterisks indicate significant differences between P_i_ and water treatment (a) or between mycorrhizal and non-mycorrhizal roots (b). (**a**) Treatment of non-inoculated plants with 5 mM KH_2_PO_4_ alone (black columns) compared to controls with water alone (white columns). Values are expressed relative to the water-treated controls that were set to 100% for each nutrient. (**b**) Nutrient content of mycorrhizal plants treated with 5 mM KH_2_PO_4_ (black columns) compared to non-inoculated controls treated with 5 mM KH_2_PO_4_ (white columns; corresponding to black columns in (a)). Values are expressed relative to the non-colonized P_i_-treated controls that were set to 100% for each nutrient.

**Figure 6:**
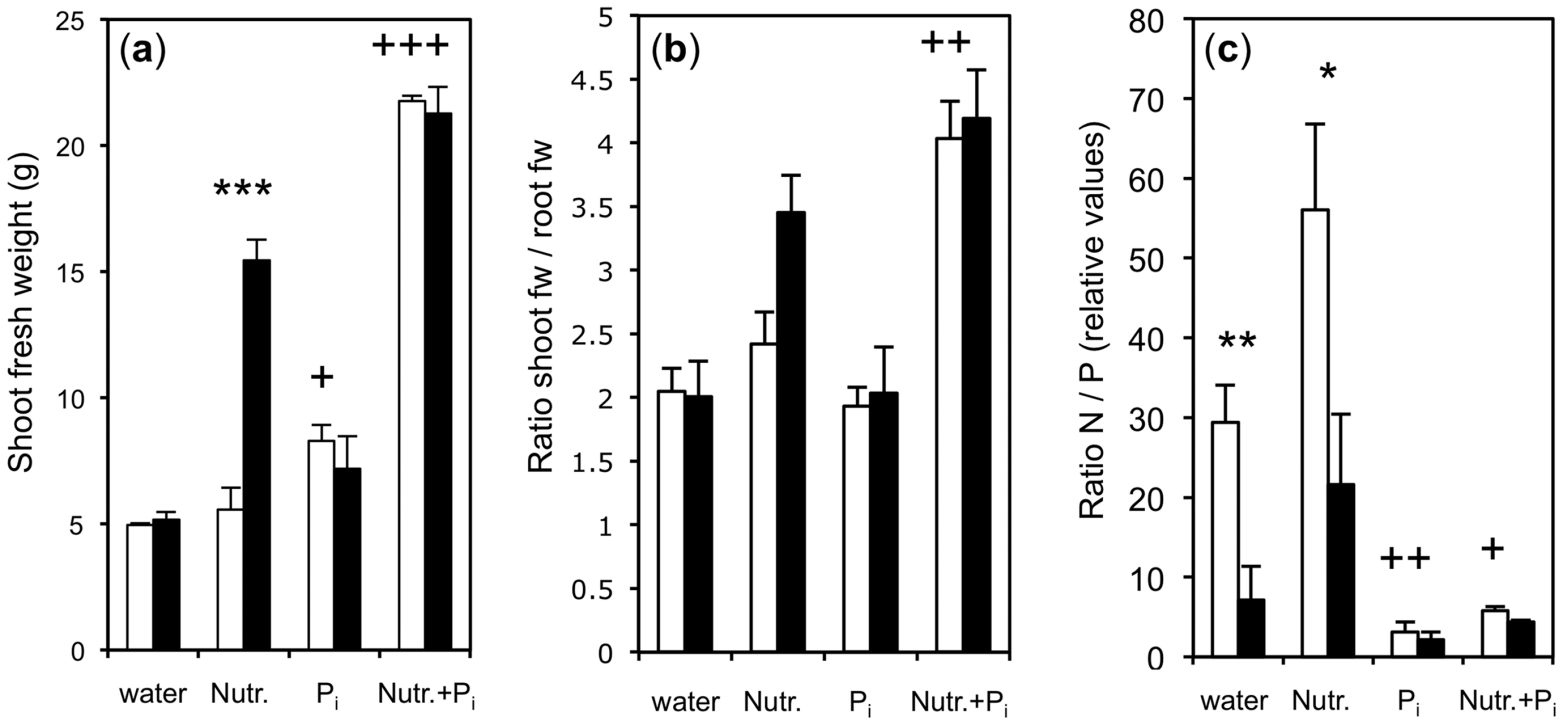
Shoot weight, shoot/root ratio and N/P ratio as indicators of nutritional status. Treatments were as in Figure 3, shown are the values of the final time point (48 days after inoculation). Columns represent the average of three biological replicates, error bars represent standard deviations. Asterisks indicate significant differences between mycorrhizal and non-mycorrhizal plants (white vs. black columns), crosses indicate significant differences between the non-mycorrhizal nutrient treatments vs. the non-mycorrhizal water treatment (i.e. between the different white columns). (a) Shoot weight of plants grown with *R. irregularis* (black column) or without (white columns) under different nutritional conditions. (b) Shoot/root ratio of plants inoculated with *R. irregularis* (black columns) or without (white columns) under various nutritional conditions. A ratio of 3.5–4 indicates that plants are well supplied with mineral nutrients, whereas a ratio around 2 indicates that plants are starved and allocate relatively large amounts of resources to the root system to compensate nutritional deficits. (c) N/P ratio of the same plants as in (a),(b). In the absence of exogenous P_i_ supply, mycorrhizal plants (black columns) exhibited lower N/P ratios than non-mycorrhizal controls, reflecting increased mycorrhizal P_i_ supply. Administration of 5 mM KH_2_PO_4_ reduced N/P ratio even stronger than AM, in particular if only P_i_ was supplied.

**Figure 7:**
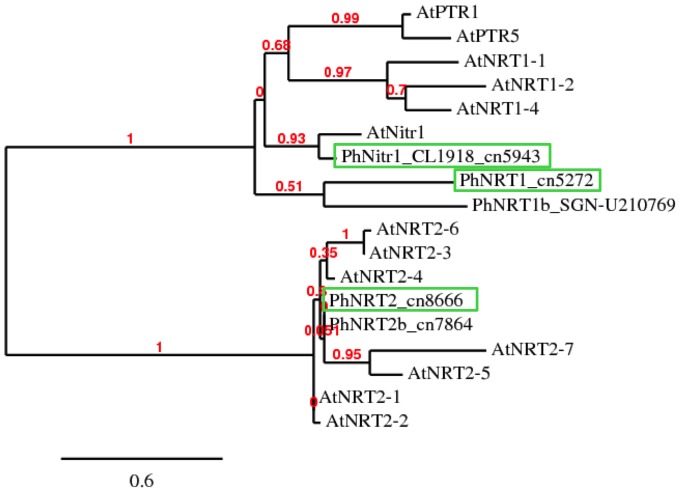
Phylogenetic analysis of P_i_-responsive transporters of *Petunia hybrida* compared to *Arabidopsis* transporters for nitrate, nitrite, and peptides. For the EST sequences listed in Table 1 the full-length predicted cDNA sequences were derived from the petunia genome sequence. Predicted petunia protein sequences were compared with *Arabidopsis thaliana* transporters for nitrate and nitrite (NRT and Nitr1, respectively), and for peptide transporters (PTR). Note the clear separation of the nitrate transporter subfamilies NRT1 and NRT2. The NRT1 family also comprises the nitrite transporter AtNitr1 and several peptide transporters, of which only two are represented (AtPTR2 and AtPTR5). Petunia has two very closely homologous representatives of the high affinity nitrate transporter family NRT2 (cn8666 and cn7864). In addition, there is a putative nitrite transporter (corresponding to the EST sequences CL1918 and cn5943), and two additional members of the low affinity NRT1 family. Genes boxed in green were analyzed by qPCR (Figure 8). Cn8665, which is almost identical to cn8666, and CL5245, which is predicted to encode a nitrogen limitation adaptation gene (see Table 1), were omitted from phylogenetic analysis.

**Figure 8:**
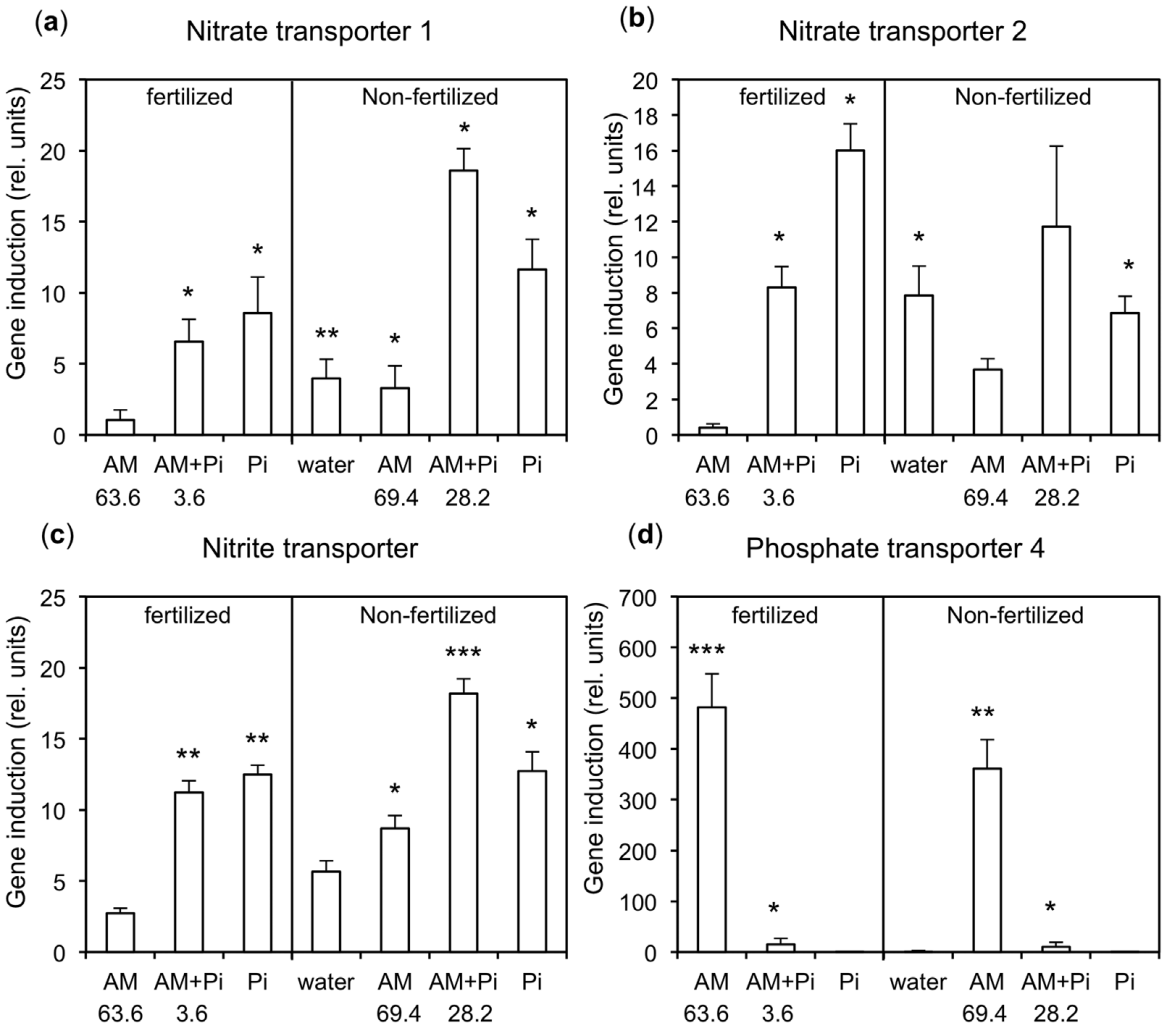
Quantitative real time PCR analysis (qPCR) of marker genes for nitrogen and phosphorus acquisition. qPCR analysis was performed to determine the expression of the N transporters boxed in Figure 7, and of the AM-specific phosphate transporter PhPT4. Treatments were as in Figures 3–6 and Table 1. Expression values were first normalized to GAPDH and then expressed as induction ratios for the indicated treatments relative to the standard treatment (non-mycorrhizal fertilized plants), as in Table 1. Columns represent the mean of five biological replicates. Error bars represent the standard deviations derived from the two standard deviations of the compared treatments (see Materials and Methods). Numbers below the x-axis reflect the percentage of root colonization in the respective sample.

**Figure 9:**
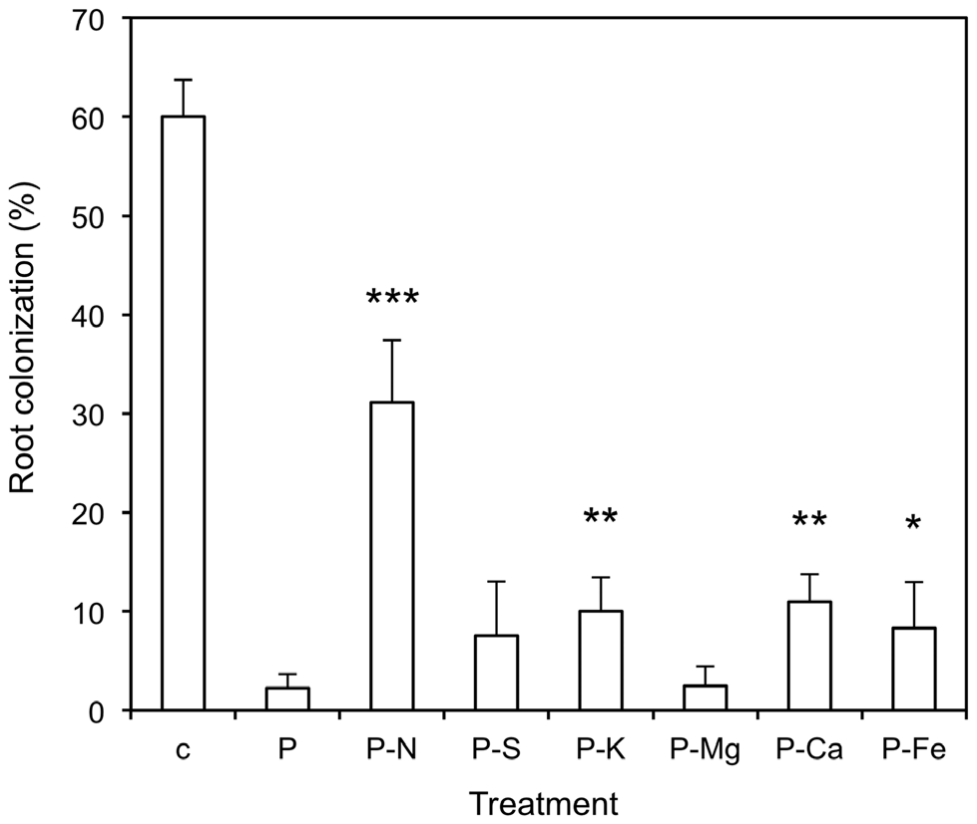
Withdrawal of individual nutrients interferes with the inhibitory effect of P_i_. Effects of withdrawal of individual nutrients from the basic nutrient solution applied together with 5_2_PO_4_. Omitted nutrients were replaced by other nutrients to maintain osmotic relationships. The strong inhibitory effect of P_i_ (P) was reduced particularly by removal of nitrate (P-N), but also to a lesser extent by removal of S, K, Ca, and Fe. The control treatment (c) represents fertilization with low P_i_ levels (0.03 mM). Columns represent the average of six biological replicates, error bars represent the standard deviations. Asterisks indicate significant differences between the treatments lacking individual nutrients and the treatment with basic nutrient solution and high P_i_.

Two experiments were carried out to assess the influence of nutrient supply on the level of AM colonization (Figures 1, 2). A third experiment was designed to assess the effect of nutrient supply and mycorrhizal colonization on plant growth, nutrient content, and gene expression (Figures 3–6, Table 1). A fourth experiment was carried out to confirm gene expression results from microarray analysis by qPCR (Figure 7,8). And a final experiment tested the effect of nutrient starvation on mycorrhizal colonization (Figure 9). All experiments were carried out with the experimental system described above. After the onset of the experiments, the plants were treated once per week for five weeks with the indicated solutions (experiments 1,2,4,5). Hence, each of these plants received a total of 250 ml of the indicated solutions over the five weeks of the experiment. In experiment 3, the plants were grown in a time course for 12 d (2 treatments), 22 d (4 treatments), 29 d (5 treatments), 36 d (6 treatments), or 48 d (7 treatments), with each treatment involving 50 ml of the respective nutrient solution. In general, the watering resulted in minimal leaching from the soil, thus the plants received the entire amount of nutrients applied to the pot. If necessary, in particular at later time points when plants had grown larger, they were watered a second time per week with only tap water to avoid water stress. Composition of the basic nutrient solution, of the modified solutions with elevated P_i_ concentration, and of the solutions lacking individual nutrients, is listed in Table S2. In the latter case, the omitted nutrients were replaced by other nutrient salts to maintain similar osmotic conditions. The effect of increasing concentrations of individual nutrients was tested with the indicated concentrations of MgSO_4_, Fe^III^-EDTA, Ca(NO_3_)_2_, and KH_2_PO_4_, respectively, in combination with basic nutrient solution.

**Table 1:** Microarray analysis of N-related transporter genes in relation to the nutritional status.

		*Fertilized plants*			*Non fertilized plants (Starved)*			
		1	2	3	4	5	6	7
		AM	AM+Pi	Pi	water	AM	AM+Pi	Pi
N/P ratio (relative to fertilized control)		0.541	0.212	0.266	0.653	0.234	0.126	0.127
Cn8666 (PhNRT2)	high-affinity nitrate transporter	1.44	18.08	21.27	18.2	11.2	16.33	37.53
CL1918 (PhNitr1)	putative nitrite transporter	7.63	13.64	10.59	6.15	26.57	27.23	23.75
cn5943 (PhNitr1)	putative nitrite transporter	6.42	19.23	15.71	6.23	41.61	41.41	17.95
cn8665	high affinity nitrate transporter	1.07	10.26	11.86	2.87	5.13	6.23	6.97
cn5272 (PhNRT1)	nitrate transporter	0.61	2.86	3.35	3.63	5.28	7.32	5.73
CL5245	nitrogen limitation adaptation	3.95	2.69	4.05	2.51	2.98	2.58	4.58
SG_SGN-U210769	nitrate transporter	1.21	1.73	1.12	1.83	4.71	10.41	3.72
cn7864	high-affinity nitrate transporter	1.32	3.01	2.96	2.83	2.69	2.85	3.51

Microarray analysis was carried out with root RNA from the plants shown in Figures 3–6 in the presence or absence of *R. irregularis* 36 d after inoculation. N/P ratio and gene expression ratios are expressed relative to reference plants supplemented with basic nutrient solution (fertilized). Relative gene induction is shown for the indicated treatments including inoculation with *R. irregularis* (AM), treatment with basic nutrient solution (fertilized plants) or water (non-fertilized plants), and treatment with 5 mM KH_2_PO_4_ (P_i_). The list represents nitrogen-related genes among the top ten in Table S4. Values represent induction ratios between expression values from the indicated treatments divided by the expression value from the reference treatment (only nutrient solution). The individual expression values and the corresponding variation coefficients are listed in Table S3.

### Determination of plant weight and AM colonization

Plants were harvested at the indicated time points to determine the fresh weight of the shoot and the root. Subsequently, root samples were taken for determination of total intraradical root colonization and, in case of experiments 3 and 4, the remaining root system was frozen in liquid N_2_ for RNA extraction. The shoots were dried (overnight at 120°C) for the determination of leaf nutrient content. The shoot:root ratio was calculated by dividing the fresh weight of the shoot by the fresh weight of the root. The mycorrhizal growth response (MGR) was calculated as the ratio of the shoot fresh weights of mycorrhizal and non-mycorrhizal plants, respectively. Data shown in Figures 3 to 6 and Table 1 come from the same experiment.

Root staining and quantification of mycorrhizal colonization were carried out as described [41]. Briefly, roots were harvested, washed and stored overnight in 10% KOH (w/v) in glass tubes. Then, they were cleared for 30 min at 95°C, washed twice with water, stained for 10 min with Trypan Blue (TB) staining solution at 95°C, and rinsed twice with 30% lactic acid (v/v). TB staining solution consisted of 20% glycerol (w/v), 30% lactic acid (v/v) and 0.01% Trypan Blue (w/v). Microscopic inspection and quantification of root colonization was carried out as described with a modified grid intersection method [41].

In general, the columns represent the mean of the values from the indicated number of biological replicates (see figure legends). Error bars represent the standard deviations. Tests for significance of differences between treatments were carried out by pairwise t-test. Significance is classified as follows: * 0.05>p>0.01, ** 0.01>p>0.001, *** p>0.001.

### Determination of metal mineral nutrients

For the quantification of K, Ca, Mg, Cu, Mn, Fe, Zn and Ni by atomic absorption spectrometry, dry leaves from time point 36 d in experiment 3 were weighed, dry ashed at 550°C for 8 h and then further processed as described [42]. Briefly, the residue was dissolved in 0.2 ml 10 M HCI and 7.8 ml water. The solutions were then mixed and diluted with 1.267 g L^−1^ CsCl suprapur in 0.1 M HCI (for potassium), with 13.37 g L^−1^ LaCl_3_-7H_2_O in 0.1 M HCI (for calcium and magnesium) and with 0.1 N HCI (for iron, manganese, copper, zinc, and nickel) prior to measuring the elements by atomic absorption spectrometry.

### Determination of reduced nitrogen in leaves

Determination of total reduced nitrogen followed a simplified version of the micro-Kjeldahl procedure reported by [43]. Leaf material (30 mg dry matter) was homogenized in 1 ml 0.01 M KOH and 200 µl of this homogenate was transferred to micro-Kjeldahl tubes, mixed with 100 µl combustion acid (4.2 M H_2_SO_4_ containing 5.4 g/l HgCl_2_) and incubated in a sand bath first at 160°C for 40 min and then for 2 h at 300°C. The samples were allowed to cool to room temperature, then 50 µl H_2_O_2_ (30%) were added, followed by further incubation at 160°C during 1 h and at 300°C for 2 h. After a second addition of 50 µl H_2_O_2_ (30%) and further incubation at 160°C for 15 min, 1 ml water was added followed by colorimetric determination of the reduced nitrogen (ammonium) by mixing 50 µl of this sample solution with 100 µl 2.5% K-Na-tartrate (w/v) and 100 µl dilute Nessler reagent (1 part of Nessler Reagent Merck Nr 109028 and two parts 2.5 M NaOH). Absorbance was measured at 450 nm.

### Determination of leaf phosphorus content

Dry leaf material (20 mg) was ashed overnight at 550°C in heat-resistant glass tubes. The residue was dissolved in 2 ml H_2_O and 100 µl HCl (32%). 1 ml solution was then transferred to Eppendorf tubes and centrifuged in a table centrifuge (12′000 rpm for 10 min). From the supernatant, 150 µl were transferred to microtiter plates, mixed with 40 µl phosphate reagent, and absorbance was measured at 405 nm. Phosphate reagent (100 ml) was prepared by mixing 20 ml ammonium heptamolybdate (5% w/v (NH_4_)_6_Mo_7_O_24_ in water), 20 ml ammonium metavanadate (2.5% w/v NH_4_VO_3_ in water), 13.8 ml concentrated nitric acid, and 46.2 ml water.

### Microarray analysis

Microarray analysis was performed as described [34]. Briefly, total root RNA from plants harvested at time point 36 d after inoculation (experiment 3) was extracted with the hot phenol procedure [44], pooled per treatment (equal amounts; n = 3), and sent to Nimblegen on dry ice. Array design of a four-plex microarray with 72′000 features was carried out using the Array-Scribe software from NimbleGen (http://www.nimblegen.com) to generate three optimized independent probes per gene, with an average length of 36 base pairs per probe. For shorter sequences, two probes per sequence were designed. Array design, probe synthesis, synthesis of labeled cDNA, hybridization, and data acquisition was carried out by Nimblegen as described [45]. Average expression values and a coefficient of variation for the gene expression values (Table S3) were derived by across-array quantile normalization using the Robust Multichip Average (RMA) algorithm [46]. Comparative analysis of expression data sets was carried out with Fire2.2 [47]. Table S3 compiles the entire data set resulting from microarray analysis. Gene expression ratios in Table 1 and Tables S4 and S5 represent the ratios of the expression values of the respective treatment divided by the expression values of the standard treatment (fertilized plants at low P_i_ levels).

### Quantitative real time RT-PCR (qPCR)

For the independent assessment of gene expression by quantitative real-time polymerase chain reaction coupled to reverse transcription (qPCR), a new experiment was carried out under identical conditions as the one represented in Table 1 and Tables S4 and S5, but in this case with five biological replicates per treatment. Total RNA was extracted using the hot phenol procedure [44]. First-strand cDNA synthesis was performed with the Omniscript RT kit according to the manufacturer's guidelines. qRT-PCR was carried out with the ABsolute qPCR SYBR Green mastermix (Thermo Scientific, http://www.thermo.com) in a Rotorgene thermocycler (Corbett Life Science, http://www.corbettlifescience.com) with the primers listed in Table S6. The PCR conditions included an initial denaturation cycle for 15 min at 95°C, followed by 40 cycles of denaturation for 15 s at 95°C, annealing for 20 s at 60°C and extension for 20 s at 72°C, followed by a final extension for 5 min at 72°C. Primer design was performed with the online primer design tool primer3 (http://bioinfo.ut.ee/primer3-0.4.0/) on the entire predicted cDNA sequence derived from the genomic sequence, which had been identified using the EST sequences. For this reason, the qPCR results (Figure 8) relate to gene names (as in the case of the phylogenetic analysis on the predicted proteins; Figure 7), and not to the array IDs. (as in Tables S4 and S5). Table 1 and Figure 7 show which array ID corresponds to which gene name.

Each treatment is represented by five biological replicate plants that were analyzed with two technical replicates each. First, the relative expression value for each gene was obtained with the delta-delta-CT method [48] using GAPDH as constitutive reference gene for normalization. Then the relative expression ratios per treatment were derived by averaging the five biological replicates +/− standard deviations. Subsequently, induction ratios were calculated between the indicated treatments relative to the reference treatment (non-mycorrhizal plants with fertilizer; see Figure 8). T-test was performed for each treatment relative to the reference treatment. Standard deviations in Figure 8 were derived using the law of propagation of the error, based on the standard deviations resulting from the five biological replicates according to the following formula (for a pair of treatments a,b): StDev = SQRT((StDev_a_/Ave_a_)^2^+ (StDev_b_/Ave_b_)^2^)*(Ave_b_/Ave_a_). Significance is classified as follows: * 0.05>p>0.01, ** 0.01>p>0.001, *** p>0.001.

### Phylogenetic sequence analysis

Predicted protein sequences were either retrieved from the public databases (*Arabidopsis thaliana*), or derived from genomic petunia sequences with the prediction tool Augustus (http://bioinf.uni-greifswald.de/augustus). The predicted amino acid sequences listed in File S1 were used for phylogenetic analysis (http://www.phylogeny.fr/version2).

## Results

### Phosphate and nitrate influence AM colonization

In order to test whether AM development is sensitive to other nutrients than phosphate (P_i_), the macronutrients nitrate, sulfate, calcium, magnesium, and the micronutrient iron were applied to petunia plants inoculated with *Rhizophagus irregularis* at elevated levels in the form of MgSO_4_, Ca(NO_3_)_2_, and Fe^III^EDTA, respectively (Figure 1). The latter was used only at sub-millimolar concentrations because of its potential toxicity [49]. Apart from P_i_ (Figure 1a), the only nutrient salt that significantly affected AM development was Ca(NO_3_)_2_ which caused a modest reduction of colonization from approximately 80% to 50% when applied at a concentration of 19 mM, whereas at 5 mM, a small enhancement of AM colonization was observed (Figure 1b). A control experiment with CaCl_2_ had no effect on AM (data not shown), suggesting that the effect of Ca(NO_3_)_2_ is caused by the nitrate anion rather than by the calcium cation. Fe^III^EDTA did not affect AM colonization (Figure 1c), and likewise, MgSO_4_ as a control for osmotic effects did not interfere with AM colonization (Figure 1d; compare also with [34]).

### Suppression of AM colonization by P_i_ depends on supply with other nutrients

To test whether the inhibition of AM development by high P_i_ depends on the nutritional context, inoculated plants were supplied with 5 mM KH_2_PO_4_ either alone or in combination with basic nutrient solution. In two additional treatments, P_i_ was supplied together with only micro- or macronutrients, respectively. While P_i_ caused a marked decrease of AM colonization in the presence of basic nutrient solution, or with only the macronutrients, inhibition reached only about 40% relative to fertilized plants (from ca. 65% colonization to ca. 25% colonization), when P_i_ was applied alone or with only micronutrients (Figure 2). Hence, the starvation for some macronutrient(s) generates an AM-promoting signal that counteracts the inhibitory effect of high P_i_.

### Dynamics of plant growth and root colonization in dependence of nutrient supply

To address the dynamics of AM colonization and plant growth in dependence of nutrient supply, a time course experiment was performed with plants treated with 5 mM KH_2_PO_4_ and nutrient solution in different combinations and in the presence and absence of *R. irregularis*. Shoot and root weight, as well as AM colonization, were determined 12, 22, 29, 36, and 48 days after inoculation. AM colonization increased most rapidly in plants treated with nutrient solution, reaching over 40% already after 22 d, whereas treatment with water resulted in generally lower colonization levels (Figure 3a). The treatment with 5 mM KH_2_PO_4_ in combination with nutrient solution strongly inhibited AM colonization, however, the inhibition was only partial in treatments with P_i_ alone.

P_i_ in combination with nutrient solution caused strong growth promotion, whereas P_i_ alone did only moderately stimulate growth (Figure 3b,c). This was the case irrespective of the presence of *R. irregularis*, indicating that under these conditions, fungal colonization did not provide a significant benefit. These results confirm that starvation for other nutrients reduces the inhibitory effect of P_i_ on AM fungal colonization (compare with Figure 2). Interestingly, a positive mycorrhizal growth response (MGR) was observed only in the presence of nutrient solution (Figure 3b-d), showing firstly that the plants could not profit from nutrient supply without the fungus, and secondly, that fungal efficiency has its limits, since plants that were not supplied with nutrient solution (and therefore relied entirely on the low nutrient concentrations in the substrate) did not show a significant MGR despite considerable colonization levels (Figure 3a,d).

### *R. irregularis* improves plant nutrition in petunia

The marked differences in AM colonization and MGR between the nutritional treatments (Figure 3) prompted us to evaluate the nutrient status of these plants as an indicator for plant fitness and qualitative mycorrhizal benefits (Figure S1). For easier comparison, the nutrient levels of the different treatments were normalized to the respective controls that were set at 100% (Figures 4, 5). First, the effect of AM on the levels of various metal ions, phosphate and nitrate in fertilized and non-fertilized plants were determined. Fertilized plants showed an AM-related increase in several mineral nutrient levels, however, only manganese and phosphate reached the significance threshold. In contrast, potassium levels were reduced in mycorrhizal roots relative to the non-mycorrhizal controls (Figure 4a). Hence, in addition to the quantitative benefit in growth (Figure 3), mycorrhizal plants profited from a qualitative improvement in mineral nutrition (in particular of phosphorus and manganese).

Similarly, plants treated only with water exhibited increased levels of calcium, zinc, and phosphorus, (Figure 4b). These results show that mycorrhizal plants profit, in addition to the mycorrhizal growth effect, from a qualitative benefit in mineral nutrition. This qualitative benefit was also observed in the absence of a positive growth response as observed in the water-treated plants.

### Exogenous P_i_ supply interferes with plant nutrition

To determine how P_i_ supply in the absence of other nutrients impacts on general plant nutrition, the nutrient levels in plants treated only with P_i_ were determined relative to plants treated with water (Figure 5a; Figure S1). As expected, supply with P_i_ alone resulted in a strong increase of P in the shoot, however, the levels of other nutrients, in particular Mg, Cu, Zn and nitrate, were reduced. This may reflect the dilution of these nutrients in the leaves as a result of the moderately stimulated growth in response to elevated P_i_.

Examination of the effect of *R. irregularis* on mineral nutrition in plants treated with P_i_ alone (without fertilization) revealed a surprising trend. The levels of several nutrients were further reduced in these mycorrhizal plants relative to the respective non-mycorrhizal controls treated with only P_i_ (Figure 5b, compare with 4a,b). This indicates that the AM fungus did not provide a nutritional benefit, but rather acted like a parasite by consuming carbohydrates, and at the same time holding back mineral nutrients. In addition, the DP for phosphate, represented by *PhPT1* and *PhPT2*, was repressed under these conditions (Tables S4, S5).

### Shoot/root ratio and N/P ratio indicate that elevated P_i_ levels cause N-starvation

Several lines of evidence indicated that P_i_-treated plants were starved for other nutrients, in particular N (see above). Therefore, three parameters that are indicative of nutritional status in plants were compared, namely shoot weight, shoot/root (S/R) ratio, and N/P ratio. Shoot growth was significantly induced by AM in fertilized plants, and by high P_i_ in combination with nutrient solution, indicating that these plants experienced favorable nutrient supply (Figure 6a). The S/R ratios (Figure 6b) showed a similar trend, in particular, the supply with high P_i_ in combination with nutrient solution caused the S/R ratio to double compared to water and P_i_ alone. Similarly, the strong growth effect of mycorrhizal plants with nutrient solution (Figure 3d) translated into a pronounced increase of S/R ratio (Figure 6b). In contrast, the treatments of water or P_i_ alone resulted in low S/R ratio even in mycorrhizal plants, indicating that these plants were nutrient-limited despite their mycorrhizal status.

Finally, the relative levels of N and P (N/P ratio) in leaves were determined in the different treatments. Normally, the N/P ratio is in the range of 10–20 [50]. Plants treated only with water had a relatively high N/P ratio of ca. 30, and the supply with nutrient solution caused the ratio to increase to ca. 55 (Figure 6c), reflecting the effect of N supply via fertilization. Under both P_i_-depleted nutrient regimes, mycorrhizal colonization dramatically reduced the N/P ratio reflecting the increased P_i_ supply through AM (Figure 6c). In this context, it is interesting to note that the improved P status in mycorrhizal plants supplied with only water did not translate into an MGR (Figure 3d), indicating that these plants were starved for nutrients other than P_i_. As expected, the treatments with high P_i_ dramatically reduced the N/P ratio in particular if P_i_ was applied alone (Figure 6c).

Taken together, these results indicate that only fertilized mycorrhizal plants and fertilized P_i_-treated plants were well supplied with nutrients, whereas the other plants were limited for some nutrients other than P_i_, conceivably N. In other words, exogenous P_i_ caused relative N-starvation. Furthermore, the N/P ratios of mycorrhizal plants suggest that AM promote P nutrition more than N nutrition.

### Microarray analysis of the P_i_ effect indicates N-starvation

In order to obtain further insight into the nutritional status of the plants, microarray analysis was employed to assess the expression of genes involved in nutrient acquisition, which can serve as a diagnostic tool. In order to reveal the consequences of elevated P_i_ levels for plant physiology, the genes that responded to a treatment with P_i_ alone (in the absence of additional fertilizer) were first identified. Among a total of 660 genes that were induced at least 3-fold by P_i_, the first ten contained three genes encoding predicted nitrate and nitrite transporters (data not shown). Focusing on only genes involved in mineral nutrient uptake revealed 28 genes induced 2-fold and 38 genes repressed 2-fold by P_i_ (Table S4). Interestingly, among the genes involved in nutrient acquisition, the ten IDs with the highest induction ratio comprised eight genes with a predicted role in nitrogen acquisition (Table 1, Table S4). Functional grouping of all P_i_-regulated genes involved in nutrient acquisition confirmed that N-related genes were induced to the highest levels, followed by sulfur-related genes, whereas all PTs were repressed (Table S5). Interestingly, no ammonium transporter responded to high P_i_.

The N-related genes comprised nitrate transporters (NRTs) of both known subfamilies (NRT1 and NRT2) and a predicted nitrite transporter (Nitr1) (File S1; Figure 7). These genes were induced by P_i_, irrespective of the presence of additional nutrients, and of the presence of the mycorrhizal fungus (Table 1, treatments 2, 3, 6, and 7), indicating that plants may experience relative N-limitation under all these conditions. In addition, they were induced in non-fertilized starved plants and, partially, in starved mycorrhizal roots (Table 1, treatments 4 and 5). This expression pattern is inversely correlated with the N/P ratio in the shoot (Table 1, first line; normalized to fertilized controls).

### Quantitative real time RT-PCR (qPCR) analysis confirms results from microarray analysis

In order to independently evaluate the expression of the marker genes for N acquisition, a new experiment under equivalent conditions was performed and the expression of genes, identified by microarray analysis, was determined by quantitative real time RT-PCR (qPCR). Representative genes for the two NRT subfamilies and for the putative Nitr1 were selected (Figure 7), and their expression compared with the expression of the functional AM marker PT4 (Figure 8). In general, the expression of the N-related genes showed a similar trend as with microarray analysis. They were strongly induced in all treatments with KH_2_PO_4_ (Figures 8a–c), and, to a lesser extent, in non-fertilized plants both, in mycorrhizal roots as well as non-mycorrhizal controls. PT4 was strongly induced in mycorrhizal plants supplied with low P_i_ levels, but only weakly in inoculated plants treated with KH_2_PO_4_ (Figure 8d). Notably, the partially restored colonization in plants treated with P_i_ alone (28.2% colonization) was not associated with a proportional restoration of PT4 induction (Figure 8d). This indicates that the MP for P_i_ remained inactive, hence, the level of AM colonization and the expression of PT4 are uncoupled under these conditions.

### Suppression of AM colonization by P_i_ depends on adequate nitrogen supply

So far, our results indicate that P caused relative N-starvation, in particular if it was applied alone (without further nutrients). Hence, it may have been the starvation for N that counteracted the inhibitory effect of high P_i_ and caused the recovery of root colonization. To test this possibility, several nutrients were removed individually by treating inoculated plants with high P_i_ together with basic nutrient solution from which individual nutrients were omitted (Figure 9; Table S2). While the treatment with all nutrients reduced AM colonization to 3%, the removal of nitrate from the nutrient solution resulted in a recovery to over 30% colonization, as in the case where all nutrients had been removed (compare with Figures 2,3). The individual removal of several other nutrient elements (S, K, Ca, Fe) also caused a partial recovery of AM colonization, although to a lesser extent.

## Discussion

### Genetic and nutritional control of AM symbiosis

During the past decade, genetic analysis of AM has revealed a suite of genes of the plant host that are essential for the establishment of the interaction and which are therefore referred to as symbiosis (SYM) genes [51]. They constitute a signaling pathway that triggers a characteristic calcium signal (calcium spiking) that is required for intracellular accommodation of the endosymbiont and for reprogramming of the host cells [51]–[53]. Apart from these endogenous factors, environmental factors influence AM in various ways. In particular, mineral nutrients are known to influence symbiosis. In general, low nutrient levels promote symbiosis, whereas high nutrient levels are inhibitory [3]. Since the primordial benefit of AM is the acquisition of mineral nutrients, the regulatory function of nutrients may represent a feedback mechanism to coordinate AM colonization with the nutritional requirements of the plant.

### Regulation of AM by phosphate

P_i_ is thought to be the major currency with which the obligate symbiotic AM fungi pay for assimilates of the plant [54], [55]. Accordingly, plants have long been known to respond particularly sensitive to high P_i_ levels with suppression of the symbiosis [30]–[38]. Inhibition of AM development by P_i_ is systemic and appears to depend on the P-status of the shoot [33], [34]. Although an active role of the plant in the P_i_-related repression of AM is plausible, the mechanisms involved are elusive.

Transcriptomic analysis of the high P_i_ response showed that besides the repression of the P_i_-starvation pathway, P_i_ repressed carotenoid biosynthetic genes and symbiosis-related genes such as PT4 [34]. Although production and secretion of the AM-promoting apocarotenoid strigolactone (SL) from the plant host is known to be inversely correlated with P_i_ supply [33], [56]–[58], reduced SL secretion alone cannot explain the inhibitory action of P_i_, since SL application does not alleviate the inhibition by P_i_ [34]. A conceivable mechanism for P-related regulation of AM is the control of fungal growth through reduced supply of assimilates to the fungus [59].

### Regulation of AM by other nutrients

For optimal growth and functioning, plants rely on balanced nutrient supply, which implies some sort of coordination between the acquisition pathways for the different nutrients. In particular, the pathways for the nutrient elements required at highest amounts, namely N and P, are known to interact [50]. The present study describes a systematic analysis of the nutritional effects of P_i_ and other nutrients on AM, and the interactions of P_i_ with other nutrients. A first outcome is the finding that among all the tested nutrients, only P_i_ and nitrate exerted a negative influence on AM, whereas other major nutrients such as potassium (K), calcium (Ca), magnesium (Mg), sulfate (SO_4_), and iron (Fe) did not influence AM at elevated concentrations (Figure 1; compare also with [34]). The relatively weak inhibitory effect of nitrate suggests that this effect may not be of central importance for the regulation of AM in the case of petunia, although a significant decline of AM colonization has been associated with high nitrogen supply in other systems [60], [61].

### Cross-talk of P with N and other nutritional pathways

Exogenous P_i_ supply in the absence of other nutrients resulted in a low S/R ratio (Figure 6b), indicative of starvation for a nutritional factor that causes the plant to prioritize root growth to compensate low nutrient supply [62]. Indeed, P_i_ supply resulted in strongly lowered N/P ratios (Figure 6c), i.e. in relative N-starvation. Nutrient starvation often leads to the induction of nutrient transporters to compensate for low nutrient supply [63]. For example, P_i_-starvation induces P_i_-transporters (PT) in *Arabidopsis* and tomato [64], [65], whereas N-starvation induces various nitrate transporters (NRT) in rice and Arabidopsis [66], [67].

Indeed, high P_i_ caused a strong induction of genes with a predicted role in N acquisition (Table 1; Tables S4 and S5), while transporters for other nutrients were affected to a lesser extent (Table S5). Interestingly, the P_i_-related induction of genes involved in N acquisition was also observed in mycorrhizal plants, in particular in the non-fertilized ones (Table 1, treatment 5; Figure 8), consistent with the decrease of the N/P ratio in these plants (Figure 6c). Among the induced NRTs (Table S5; Figure 7), three very similar genes encoded predicted high-affinity transporters within the NRT2 family (cn8665, cn8666 and cn7864), with cn8665 and cn8666 being 100% identical at the amino acid level. Hence, only cn8666 was analyzed phylogenetically as a representative of this group (Figure 7). Cn8666 and cn7864 are particularly closely related to AtNRT2.1, which is essential for the response to nitrate starvation in the roots of *Arabidopsis* [68], and to AtNRT2.4, which is induced by N-starvation in the root epidermis and contributes to nitrate acquisition under this condition [67]. Three further genes, represented by the EST sequences cn5272, cn5943, CL1918, and SG_SGN-U210769, fall into the NRT1 family (Figure 7). Besides low-affinity nitrate transporters, the NRT1 family of *Arabidospsis* contains a predicted plastidial nitrite transporter (AtNitr1) [69], and a so-called nitrate transceptor [70], [71], which is involved in both, the transport and the perception and signaling of nitrate [72]. The petunia EST consensus sequences cn5943 and CL1918 belong to the same petunia gene, which is most closely related to the *Arabidopsis* Nitr1 [69]. While several genes involved in N acquisition were induced by N starvation, two predicted nitrate transporters (cn8317 and cn8506) behaved in the opposite way, i.e. they were repressed by N starvation (Table S4 and S5). These genes may encode nitrate transporters that are induced by their own substrate, nitrate [73], [74]. Taken together, these results indicate that P_i_ induces the entire nitrate acquisition pathway, and possibly nitrate sensing in petunia.

Besides the induction of nitrate transporters that are involved in the DP for N uptake, N starvation caused AM colonization to be partially restored under high P_i_ levels (Figures 3, 6, 9), conceivably to enable symbiotic N uptake despite the inhibitory levels of P_i_. Interestingly, it has been observed in a natural environment as well that the repression of AM colonization by high P_i_ was relieved when N became limiting [40]. A similar interaction between P and N nutrition was shown on colonization of *Allium schoenoprasum* by *Glomus caledonium* in pot cultures [75]. These results are in line with the hypothesis that AM symbiosis evolved to ensure balanced nutrient levels in both partners and a stoichiometric relationship in the exchange of C, N, and P [76].

If AM colonization at high P_i_ levels was promoted to enable symbiotic N acquisition, ammonium transporters (AMT) of the plant would be expected to become induced to allow N from the fungus to be taken up in the form of ammonium [16]. However, no AMT was induced by high P_i_, indicating that despite the restoration of root colonization, the symbiosis does not contribute to N uptake under these conditions, an assumption that is in line with the reduced N levels in these plants (Figure 5b). Surprisingly, the recovery of AM colonization observed in plants supplied with high P_i_ alone was not associated with a parallel induction of symbiotic PT expression (Figure 8d), although the colonization appeared morphologically normal. PT4 is commonly regarded as a marker for functional symbiotic cells with arbuscules, however, repression of PT4 by P_i_ appears to be stronger than induction of PT4 by the AM fungus. Taken together, these findings suggest that AM colonization under these conditions does not confer an obvious nutritional benefit to the plant host.

### Inhibition of AM development by high P_i_ and by mutation of P_i_ transporters: common mechanisms?

Interestingly, mutation of AM-related PTs in the plant causes a similar inhibitory effect on AM as high exogenous P_i_ supply [77]–[79]. However, in these cases, it is not the abundant supply, but the lack of P_i_ delivery that triggers inhibition. A possible explanation for this observation is that the plant can assess the costs (assimilate consumption of the fungus), and the benefits (fungal P_i_ supply) in the interaction, and inhibits fungal colonization if the balance is unfavorable. The fact that P_i_ strongly represses symbiotic PTs (Table S5) and other symbiosis-related genes [34] raises the possibility that the high P_i_ effect and the PT mutant phenotype may involve partially overlapping mechanisms: Although the two conditions have opposite consequences for the P status of the plant, they both result in inhibition of the symbiotic P_i_ uptake pathway.

Inhibition of AM development by PT mutation has been observed in legumes and rice [77]–[79]. Mutation of a close homologue in tomato had no AM-related phenotype [80], [81], conceivably because of functional redundancy among the three symbiotic PT genes commonly found in *Solanaceae* [81]–[83]. In *P. hybrida*, all three PTs were strongly repressed by P_i_, thus resulting in a general conditional inactivation of the symbiotic P_i_ uptake pathway [34], potentially contributing to inhibition of AM development as in PT mutants of legumes and rice. An interesting parallel concerning the inhibitory effects of high P_i_ and of PT mutation on AM development extends to the fact that both conditions can be reversed by N-starvation (Figure 9; Javot et al., 2011).

### Mycorrhizal benefits depend on nutrient supply

The primary benefit of AM is thought to be the improved supply of macro- and micronutrients [21], [84], in particular of P_i_, which translates into increased growth rates of mycorrhizal plants, the so-called mycorrhizal growth response (MGR). Indeed, a robust MGR and enhanced nutrient levels in mycorrhizal plants were observed upon fertilization with basic nutrient solution (Figures 3d, 4a; Figure S1). Taking into account both the MGR and the increased nutrient content per gram of leaf tissue, mycorrhizal plants contained approximately 6-fold the amount of P_i_ compared to fertilized plants without AM, and approximately 4-fold the amount of the other nutrients.

Unfertilized mycorrhizal plants also had increased nutrient levels compared to non-mycorrhizal control plants, although to a lesser extent, and only P_i_ accumulation was substantial (Figure 4b), showing that despite the lack of an MGR (Figures 2, 3), these plants had gained a qualitative benefit from AM symbiosis. Although the growth of these plants was apparently limited by some factor, the higher nutrient content represents a potential advantage in fitness that can be translated into growth or in the production of additional offspring at a later stage of development.

On the other end of the scale, plants treated with only P_i_ alone were colonized to 30% but had no positive MGR and no induction of the symbiotic P_i_ uptake machinery. Notably, these plants even contained lower nutrient levels than non-mycorrhizal control plants treated with P_i_ only, hence, under these conditions, the interaction exhibited characteristics of a parasitic interaction.

MGR varies considerably with some plant species showing spectacular increases of >10-fold. Such cases are usually based on the bad performance of the plant in the absence of the fungal partner, a phenomenon that is expressed as ‘dependency’ of the plant on AM [85]. In contrast, many species, in particular cereals, show no (or even negative) MGR, and therefore are considered non-responsive [85], [86]. However, it has been pointed out that such classifications represent an oversimplification [26], since the outcome of an interaction depends on both partners and involves environmental factors [87]. Indeed, our study shows that the environment has a strong influence on the MGR of *Petunia hybrida* with *R. irregularis*. Although plants in natural environments may experience less extreme conditions than in our experiments, our results show that depending on the nutritional conditions, an AM interaction can represent a range of outcomes along the continuum between strongly mutualistic and parasitic [88]. However, it should be kept in mind that mycorrhizal plants with neutral or negative MGR may profit from other benefits than improved nutrition, for example increased drought tolerance or disease resistance [7]–[9].

## Conclusions

Nutrient-dependent regulation of AM colonization provides an important feedback mechanism for plants to promote or limit fungal colonization according to their needs. We show here that the nutrients P_i_ and nitrate can potentially exert negative regulation on AM, whereas sulfate and the cations Mg^2+^, Ca^2+^, and Fe^3+^ have no effect. On the other hand, starvation for several mineral nutrients, in particular for nitrate, reversed the inhibitory effect of P_i_ on AM, indicating that nutrient starvation triggers a dominant AM-promoting signal that counteracts the effects of high P_i_. Future research should address the interplay of exogenous and endogenous factors in AM, in particular, how nutrients impinge on symbiotic signaling and on the subsequent cellular program in host cells.

## Supporting Information

Figure S1 **Nutrient content of mycorrhizal and non-mycorrhizal plants under different nutritional treatments.** Nutrient levels were determined in the leaves of plants treated with only water, with basic nutrient solution (Nutr.), with 5 mM KH_2_PO_4_ (P), or with a combination of nutrient solution and 5 mM KH_2_PO_4_ (Nutr.+P). Plants were harvested 36 days after inoculation with *R. irregularis* (black columns) or mock inoculation (white columns). Columns represent the average of three biological replicates, error bars represent the standard deviations.
(TIF)

File S1 **Predicted amino acid sequences of nitrate and nitrite transporters of** ***Arabidopsis thaliana*** **and** ***Petunia hybrida*** **used for phylogenetic analysis.**
(DOCX)

Table S1 Nutrient content of plant growth substrate.
(XLSX)

Table S2 Composition of nutrient solutions.
(XLSX)

Table S3 Microarray data after first level analysis by Nimblegen. Mean gene expression values (Exprs) and variation coefficients (SE_Exprs) resulting from the three probes per sequence ID are listed for the different nutritional regimes. All the induction ratios shown in Tables S4 and S5, and Table 1, are derived from the data listed in Table S3.
(XLSX)

Table S4 Genes regulated by high P_i_ supply. Gene expression ratios above 2-fold and below 0.5-fold of genes with a predicted role in mineral nutrient acquisition are shown. Expression ratios were obtained by dividing the expression values from the treatment of interest (indicated combinations of basic nutrient solution, 5 mM KH_2_PO_4_, and *R. irregularis*) by the expression values of the reference treatment, i.e. non-mycorrhizal fertilized plants (see Table S3 for individual expression values). Expression ratios were sorted according to the treatment with high P_i_ alone (treatment 7). Note that the ten genes induced at highest levels comprise eight N-related genes (in bold), in particular transporters (see also Table 1).
(XLS)

Table S5 Nutritional regulation of genes involved in nutrient acquisition. Expression ratios as in Table S4 but ordered according to predicted function.
(XLS)

Table S6 Primers for quantitative real time RT-PCR (qPCR).
(XLSX)

## References

[1] Elser JJ, Bracken MES, Cleland EE, Gruner DS, Harpole WS, et al (2007) Global analysis of nitrogen and phosphorus limitation of primary producers in freshwater, marine and terrestrial ecosystems. Ecology Letters 10: 1135–1142. 17922835 10.1111/j.1461-0248.2007.01113.x

[2] Hell R, Hillebrand H (2001) Plant concepts for mineral acquisition and allocation. Current Opinion in Biotechnology 12: 161–168. 11287231 10.1016/s0958-1669(00)00193-2

[3] Smith S, Read D (2008) Mycorrhizal Symbiosis. New York: Academic Press.

[4] Brundrett MC (2002) Coevolution of roots and mycorrhizas of land plants. New Phytologist 154: 275–304. 10.1046/j.1469-8137.2002.00397.x 33873429

[5] Friese CF, Allen MF (1991) The spread of VA mycorrhizal fungal hyphae in the soil – Inoculum types and external hyphal architecture. Mycologia 83: 409–418.

[6] Bago B, Azcon-Aguilar C, Goulet A, Piche Y (1998) Branched absorbing structures (BAS): a feature of the extraradical mycelium of symbiotic arbuscular mycorrhizal fungi. New Phytologist 139: 375–388.

[7] Augé RM (2001) Water relations, drought and vesicular-arbuscular mycorrhizal symbiosis. Mycorrhiza 11: 3–42.

[8] Conrath U, Beckers GJM, Flors V, Garcia-Agustin P, Jakab G, et al (2006) Priming: Getting ready for battle. Molecular Plant-Microbe Interactions 19: 1062–1071. 17022170 10.1094/MPMI-19-1062

[9] Jung SC, Martinez-Medina A, Lopez-Raez JA, Pozo MJ (2012) Mycorrhiza-induced resistance and priming of plant defenses. Journal of Chemical Ecology 38: 651–664. 22623151 10.1007/s10886-012-0134-6

[10] Karandashov V, Bucher M (2005) Symbiotic phosphate transport in arbuscular mycorrhizas. Trends in Plant Science 10: 22–29. 15642520 10.1016/j.tplants.2004.12.003

[11] Fellbaum CR, Gachomo EW, Beesetty Y, Choudhari S, Strahan GD, et al (2012) Carbon availability triggers fungal nitrogen uptake and transport in arbuscular mycorrhizal symbiosis. Proceedings of the National Academy of Sciences of the United States of America 109: 2666–2671. 22308426 10.1073/pnas.1118650109 PMC3289346

[12] Hodge A, Fitter AH (2010) Substantial nitrogen acquisition by arbuscular mycorrhizal fungi from organic material has implications for N cycling. Proceedings of the National Academy of Sciences of the United States of America 107: 13754–13759. 20631302 10.1073/pnas.1005874107 PMC2922220

[13] Mäder P, Vierheilig H, Streitwolf-Engel R, Boller T, Frey B, et al (2000) Transport of N-15 from a soil compartment separated by a polytetrafluoro-ethylene membrane to plant roots via the hyphae of arbuscular mycorrhizal fungi. New Phytologist 146: 155–161.

[14] Müller A, George E, Gabriel-Neumann E (2013) The symbiotic recapture of nitrogen from dead mycorrhizal and non-mycorrhizal roots of tomato plants. Plant and Soil 364: 341–355.

[15] Govindarajulu M, Pfeffer PE, Jin HR, Abubaker J, Douds DD, et al (2005) Nitrogen transfer in the arbuscular mycorrhizal symbiosis. Nature 435: 819–823. 15944705 10.1038/nature03610

[16] Guether M, Neuhauser B, Balestrini R, Dynowski M, Ludewig U, et al (2009) A mycorrhizal-specific ammonium transporter from *Lotus japonicus* acquires nitrogen released by arbuscular mycorrhizal fungi. Plant Physiology 150: 73–83. 19329566 10.1104/pp.109.136390 PMC2675747

[17] Tian CJ, Kasiborski B, Koul R, Lammers PJ, Bucking H, et al (2010) Regulation of the nitrogen transfer pathway in the arbuscular mycorrhizal symbiosis: Gene characterization and the coordination of expression with nitrogen flux. Plant Physiology 153: 1175–1187. 20448102 10.1104/pp.110.156430 PMC2899933

[18] Allen JW, Shachar-Hill Y (2009) Sulfur transfer through an arbuscular mycorrhiza. Plant Physiology 149: 549–560. 18978070 10.1104/pp.108.129866 PMC2613693

[19] Sieh D, Watanabe M, Devers EA, Brueckner F, Hoefgen R, et al (2013) The arbuscular mycorrhizal symbiosis influences sulfur starvation responses of Medicago truncatula. New Phytologist 197: 606–616. 23190168 10.1111/nph.12034

[20] Clark RB, Zeto SK (2000) Mineral acquisition by arbuscular mycorrhizal plants. Journal of Plant Nutrition 23: 867–902.

[21] George E (2000) Nutrient uptake – Contributions of arbuscular mycorrhizal fungi to plant mineral nutrition. In: Kapulnik Y, Douds DD, editors. Arbuscular mycorrhizas: Physiology and function. Dordrecht: Kluwer Academic Publishers. 307–343.

[22] Smith SE, Smith FA (2011) Roles of arbuscular mycorrhizas in plant nutrition and growth: New paradigms from cellular to ecosystem scales. Annual Review of Plant Biology 62: 227–250. 10.1146/annurev-arplant-042110-103846 21391813

[23] Smith SE, Smith FA, Jakobsen I (2003) Mycorrhizal fungi can dominate phosphate supply to plants irrespective of growth responses. Plant Physiology 133: 16–20. 12970469 10.1104/pp.103.024380 PMC1540331

[24] Smith SE, Smith FA, Jakobsen I (2004) Functional diversity in arbuscular mycorrhizal (AM) symbioses: the contribution of the mycorrhizal P uptake pathway is not correlated with mycorrhizal responses in growth or total P uptake. New Phytologist 162: 511–524.

[25] Klironomos JN (2003) Variation in plant response to native and exotic arbuscular mycorrhizal fungi. Ecology 84: 2292–2301.

[26] Smith FA, Grace EJ, Smith SE (2009) More than a carbon economy: nutrient trade and ecological sustainability in facultative arbuscular mycorrhizal symbioses. New Phytologist 182: 347–358. 19207688 10.1111/j.1469-8137.2008.02753.x

[27] Alguacil MM, Lumini E, Roldan A, Salinas-Garcia JR, Bonfante P, et al (2008) The impact of tillage practices on arbuscular mycorrhizal fungal diversity in subtropical crops. Ecological Applications 18: 527–536. 18488613 10.1890/07-0521.1

[28] Lin XG, Feng YZ, Zhang HY, Chen RR, Wang JH, et al (2012) Long-term balanced fertilization decreases arbuscular mycorrhizal fungal diversity in an arable soil in north China revealed by 454 pyrosequencing. Environmental Science & Technology 46: 5764–5771. 22582875 10.1021/es3001695

[29] Liu YJ, Shi GX, Mao L, Cheng G, Jiang SJ, et al (2012) Direct and indirect influences of 8 yr of nitrogen and phosphorus fertilization on Glomeromycota in an alpine meadow ecosystem. New Phytologist 194: 523–535. 22292929 10.1111/j.1469-8137.2012.04050.x

[30] Abbott LK, Robson AD, De Boer G (1984) The effect of phosphorus on the formation of hyphae in soil by the vesicular arbuscular mycorrhizal fungus, *Glomus fasciculatum* . New Phytologist 97: 437–446.

[31] Amijée F, Tinker PB, Stribley DP (1989) Effects of phosphorus on the morphology of VA mycorrhizal root-system of leek (*Allium porrum* L). Plant and Soil 119: 334–336.

[32] Amijée F, Tinker PB, Stribley DP (1989) The development of endomycorrhizal root systems .7. A detailed study of effects of soil-phosphorus on colonization. New Phytologist 111: 435–446. 10.1111/j.1469-8137.1989.tb00706.x 33874001

[33] Balzergue C, Puech-Pagès V, Bécard G, Rochange SF (2011) The regulation of arbuscular mycorrhizal symbiosis by phosphate in pea involves early and systemic signalling events. Journal of Experimental Botany 62: 1049–1060. 21045005 10.1093/jxb/erq335 PMC3022399

[34] Breuillin F, Schramm J, Hajirezaei M, Ahkami A, Favre P, et al (2010) Phosphate systemically inhibits development of arbuscular mycorrhiza in *Petunia hybrida* and represses genes involved in mycorrhizal functioning. Plant Journal 64: 1002–1017. 21143680 10.1111/j.1365-313X.2010.04385.x

[35] Hepper CM (1983) The effect of nitrate and phosphate on the vesicular arbuscular mycorrhizal infection of lettuce. New Phytologist 93: 389–399.

[36] Jasper DA, Robson AD, Abbott LK (1979) Phosphorus and the formation of vesicular-arbuscular mycorrhizas. Soil Biology & Biochemistry 11: 501–505.

[37] Menge JA, Steirle D, Bagyaraj DJ, Johnson ELV, Leonard RT (1978) Phosphorus concentrations in plants responsible for inhibition of mycorrhizal infection. New Phytologist 80: 575–578.

[38] Thomson BD, Robson AD, Abbott LK (1986) Effects of phosphorus on the formation of mycorrhizas by Gigaspora calospora and Glomus fasciculatum in relation to root carbohydrates. New Phytologist 103: 751–765.

[39] Douds DD, Pfeffer PE, Shachar-Hill Y (2000) Carbon partitioning, cost, and metabolism of arbuscular mycorrhizas. In: Kapulnik Y, Douds DD, editors. Arbuscular Mycorrhizas: Physiology and Function. Dordrecht: Kluwer Academic Publishers.

[40] Blanke V, Renker C, Wagner M, Fullner K, Held M, et al (2005) Nitrogen supply affects arbuscular mycorrhizal colonization of Artemisia vulgaris in a phosphate-polluted field site. New Phytologist 166: 981–992. 15869657 10.1111/j.1469-8137.2005.01374.x

[41] Sekhara Reddy DMR, Schorderet M, Feller U, Reinhardt D (2007) A petunia mutant affected in intracellular accommodation and morphogenesis of arbuscular mycorrhizal fungi. Plant Journal 51: 739–750. 17573800 10.1111/j.1365-313X.2007.03175.x

[42] Stieger PA, Feller U (1994) Nutrient accumulation and translocation in maturing wheat plants grown on waterlogged soil. Plant and Soil 160: 87–95.

[43] Bohley P (1967) Reihenbestimmungen von Stickstoff im Ultramikromassstab – Kjeldahlveraschung und Phenol-Hypochlorit-Reaktion. Hoppe-Seylers Zeitschrift für Physiologische Chemie 348: 100–110. 5592395

[44] Verwoerd TC, Dekker BMM, Hoekema A (1989) A small-scale procedure for the rapid isolation of plant RNAs. Nucleic Acids Research 17: 2362–2362. 2468132 10.1093/nar/17.6.2362 PMC317610

[45] Nuwaysir EF, Huang W, Albert TJ, Singh J, Nuwaysir K, et al (2002) Gene expression analysis using oligonucleotide arrays produced by maskless photolithography. Genome Research 12: 1749–1755. 12421762 10.1101/gr.362402 PMC187555

[46] Irizarry RA, Hobbs B, Collin F, Beazer-Barclay YD, Antonellis KJ, et al (2003) Exploration, normalization, and summaries of high density oligonucleotide array probe level data. Biostatistics 4: 249–264. 12925520 10.1093/biostatistics/4.2.249

[47] Garcion C, Metraux JP (2006) FiRe and microarrays: a fast answer to burning questions. Trends in Plant Science 11: 320–322. 16807061 10.1016/j.tplants.2006.05.009

[48] Schmittgen TD, Livak KJ (2008) Analyzing real-time PCR data by the comparative C-T method. Nature Protocols 3: 1101–1108. 18546601 10.1038/nprot.2008.73

[49] Nagajyoti PC, Lee KD, Sreekanth TVM (2010) Heavy metals, occurrence and toxicity for plants: a review. Environmental Chemistry Letters 8: 199–216.

[50] Güsewell S (2004) N:P ratios in terrestrial plants: variation and functional significance. New Phytologist 164: 243–266. 10.1111/j.1469-8137.2004.01192.x 33873556

[51] Parniske M (2008) Arbuscular mycorrhiza: the mother of plant root endosymbioses. Nature Reviews Microbiology 6: 763–775. 18794914 10.1038/nrmicro1987

[52] Charpentier M, Oldroyd GE (2013) Nuclear calcium signaling in plants. Plant Physiology 163: 496–503. 23749852 10.1104/pp.113.220863 PMC3793031

[53] Seddas P, Gianinazzi-Pearson V, Schoefs B, Kuster H, Wipf D (2009) Communication and signaling in the plant-fungus symbiosis: The mycorrhiza. In: Baluska F, editor. Plant-Environment Interactions: From Sensory Plant Biology to Active Plant Behavior. 45–71.

[54] Javot H, Pumplin N, Harrison MJ (2007) Phosphate in the arbuscular mycorrhizal symbiosis: transport properties and regulatory roles. Plant, Cell and Environment 30: 310–322. 10.1111/j.1365-3040.2006.01617.x 17263776

[55] Bucher M (2007) Functional biology of plant phosphate uptake at root and mycorrhiza interfaces. New Phytologist 173: 11–26. 17176390 10.1111/j.1469-8137.2006.01935.x

[56] Lopez-Raez JA, Charnikhova T, Gomez-Roldan V, Matusova R, Kohlen W, et al (2008) Tomato strigolactones are derived from carotenoids and their biosynthesis is promoted by phosphate starvation. New Phytologist 178: 863–874. 18346111 10.1111/j.1469-8137.2008.02406.x

[57] Yoneyama K, Yoneyama K, Takeuchi Y, Sekimoto H (2007) Phosphorus deficiency in red clover promotes exudation of orobanchol, the signal for mycorrhizal symbionts and germination stimulant for root parasites. Planta 225: 1031–1038. 17260144 10.1007/s00425-006-0410-1

[58] Yoneyama K, Xie XN, Kusumoto D, Sekimoto H, Sugimoto Y, et al (2007) Nitrogen deficiency as well as phosphorus deficiency in sorghum promotes the production and exudation of 5-deoxystrigol, the host recognition signal for arbuscular mycorrhizal fungi and root parasites. Planta 227: 125–132. 17684758 10.1007/s00425-007-0600-5

[59] Olsson PA, Rahm J, Aliasgharzad N (2010) Carbon dynamics in mycorrhizal symbioses is linked to carbon costs and phosphorus benefits. FEMS Microbiology Ecology 72: 123–131. 10.1111/j.1574-6941.2009.00833.x 20459516

[60] Egerton-Warburton LM, Graham RC, Allen EB, Allen MF (2001) Reconstruction of the historical changes in mycorrhizal fungal communities under anthropogenic nitrogen deposition. Proceedings of the Royal Society B-Biological Sciences 268: 2479–2484. 10.1098/rspb.2001.1844 PMC1088903 11747567

[61] van Diepen LTA, Lilleskov EA, Pregitzer KS, Miller RM (2007) Decline of arbuscular mycorrhizal fungi in northern hardwood forests exposed to chronic nitrogen additions. New Phytologist 176: 175–183. 17803648 10.1111/j.1469-8137.2007.02150.x

[62] Hermans C, Hammond JP, White PJ, Verbruggen N (2006) How do plants respond to nutrient shortage by biomass allocation? Trends in Plant Science 11: 610–617. 17092760 10.1016/j.tplants.2006.10.007

[63] Amtmann A, Blatt MR (2009) Regulation of macronutrient transport. New Phytologist 181: 35–52. 19076716 10.1111/j.1469-8137.2008.02666.x

[64] Muchhal US, Pardo JM, Raghothama KG (1996) Phosphate transporters from the higher plant Arabidopsis thaliana. Proceedings of the National Academy of Sciences of the United States of America 93: 10519–10523. 8927627 10.1073/pnas.93.19.10519 PMC38418

[65] Muchhal US, Raghothama KG (1999) Transcriptional regulation of plant phosphate transporters. Proceedings of the National Academy of Sciences of the United States of America 96: 5868–5872. 10318976 10.1073/pnas.96.10.5868 PMC21952

[66] Cai HM, Lu YG, Xie WB, Zhu T, Lian XM (2012) Transcriptome response to nitrogen starvation in rice. Journal of Biosciences 37: 731–747. 22922198 10.1007/s12038-012-9242-2

[67] Kiba T, Feria-Bourrellier AB, Lafouge F, Lezhneva L, Boutet-Mercey S, et al (2012) The Arabidopsis nitrate transporter NRT2.4 plays a double role in roots and shoots of nitrogen-starved plants. Plant Cell 24: 245–258. 22227893 10.1105/tpc.111.092221 PMC3289576

[68] Remans T, Nacry P, Pervent M, Girin T, Tillard P, et al (2006) A central role for the nitrate transporter NRT2.1 in the integrated morphological and physiological responses of the root system to nitrogen limitation in Arabidopsis. Plant Physiology 140: 909–921. 16415211 10.1104/pp.105.075721 PMC1400583

[69] Sugiura M, Georgescu MN, Takahashi M (2007) A nitrite transporter associated with nitrite uptake by higher plant chloroplasts. Plant and Cell Physiology 48: 1022–1035. 17566055 10.1093/pcp/pcm073

[70] Bouguyon E, Gojon A, Nacry P (2012) Nitrate sensing and signaling in plants. Seminars in Cell & Developmental Biology 23: 648–654. 22273693 10.1016/j.semcdb.2012.01.004

[71] Gojon A, Krouk G, Perrine-Walker F, Laugier E (2011) Nitrate transceptor(s) in plants. Journal of Experimental Botany 62: 2299–2308. 21239382 10.1093/jxb/erq419

[72] Wang RC, Xing XJ, Wang Y, Tran A, Crawford NM (2009) A genetic screen for nitrate regulatory mutants captures the nitrate transporter gene NRT1.1. Plant Physiology 151: 472–478. 19633234 10.1104/pp.109.140434 PMC2735993

[73] Dechorgnat J, Nguyen CT, Armengaud P, Jossier M, Diatloff E, et al (2011) From the soil to the seeds: the long journey of nitrate in plants. Journal of Experimental Botany 62: 1349–1359. 21193579 10.1093/jxb/erq409

[74] Tsay YF, Chiu CC, Tsai CB, Ho CH, Hsu PK (2007) Nitrate transporters and peptide transporters. FEBS Letters 581: 2290–2300. 17481610 10.1016/j.febslet.2007.04.047

[75] Baath E, Spokes J (1989) The effect of added nitrogen and phosphorus on mycorrhizal growth-response and infection in *Allium schoenoprasum* . Canadian Journal of Botany 67: 3227–3232.

[76] Johnson NC (2010) Resource stoichiometry elucidates the structure and function of arbuscular mycorrhizas across scales. New Phytologist 185: 631–647. 19968797 10.1111/j.1469-8137.2009.03110.x

[77] Javot H, Penmetsa RV, Terzaghi N, Cook DR, Harrison MJ (2007) A *Medicago truncatula* phosphate transporter indispensable for the arbuscular mycorrhizal symbiosis. Proceedings of the National Academy of Sciences of the United States of America 104: 1720–1725. 17242358 10.1073/pnas.0608136104 PMC1785290

[78] Maeda D, Ashida K, Iguchi K, Chechetka SA, Hijikata A, et al (2006) Knockdown of an arbuscular mycorrhiza-inducible phosphate transporter gene of Lotus japonicus suppresses mutualistic symbiosis. Plant and Cell Physiology 47: 807–817. 16774930 10.1093/pcp/pcj069

[79] Yang SY, Gronlund M, Jakobsen I, Grotemeyer MS, Rentsch D, et al (2012) Nonredundant regulation of rice arbuscular mycorrhizal symbiosis by two members of the PHOSPHATE TRANSPORTER1 gene family. Plant Cell 24: 4236–4251. 23073651 10.1105/tpc.112.104901 PMC3517247

[80] Nacry P, Canivenc G, Muller B, Azmi A, Van Onckelen H, et al (2005) A role for auxin redistribution in the responses of the root system architecture to phosphate starvation in Arabidopsis. Plant Physiol 138: 2061–2074. 16040660 10.1104/pp.105.060061 PMC1183395

[81] Nagy F, Karandashov V, Chague W, Kalinkevich K, Tamasloukht M, et al (2005) The characterization of novel mycorrhiza-specific phosphate transporters from *Lycopersicon esculentum* and *Solanum tuberosum* uncovers functional redundancy in symbiotic phosphate transport in solanaceous species. Plant Journal 42: 236–250. 15807785 10.1111/j.1365-313X.2005.02364.x

[82] Chen A, Hu J, Sun S, Xu G (2007) Conservation and divergence of both phosphate- and mycorrhiza-regulated physiological responses and expression patterns of phosphate transporters in solanaceous species. New Phytologist 173: 817–831. 17286830 10.1111/j.1469-8137.2006.01962.x

[83] Wegmüller S, Svistoonoff S, Reinhardt D, Stuurman J, Amrhein N, et al (2008) A transgenic dTph1 insertional mutagenesis system for forward genetics in mycorrhizal phosphate transport of Petunia. Plant Journal 54: 1115–1127. 18315538 10.1111/j.1365-313X.2008.03474.x

[84] Clark RB, Zeto SK (1996) Mineral acquisition by mycorrhizal maize grown on acid and alkaline soil. Soil Biology & Biochemistry 28: 1495–1503.

[85] Tawaraya K (2003) Arbuscular mycorrhizal dependency of different plant species and cultivars. Soil Science and Plant Nutrition 49: 655–668.

[86] Angelard C, Colard A, Niculita-Hirzel H, Croll D, Sanders IR (2010) Segregation in a mycorrhizal fungus alters rice growth and symbiosis-specific gene transcription. Current Biology 20: 1216–1221. 20541408 10.1016/j.cub.2010.05.031

[87] Sawers RJH, Gebreselassie MN, Janos DP, Paszkowski U (2010) Characterizing variation in mycorrhiza effect among diverse plant varieties. Theoretical and Applied Genetics 120: 1029–1039. 20012933 10.1007/s00122-009-1231-y

[88] Johnson NC, Graham JH, Smith FA (1997) Functioning of mycorrhizal associations along the mutualism−parasitism continuum. New Phytologist 135: 575–586.

