# Correction: Phosphorus and Nitrogen Regulate Arbuscular Mycorrhizal Symbiosis in *Petunia hybrida*

**DOI:** 10.1371/journal.pone.0127472

**Published:** 2015-04-29

**Authors:** Eva Nouri, Florence Breuillin-Sessoms, Urs Feller, Didier Reinhardt

The second group heading in column one of S2 Table contains the wrong unit. It should read: Microelements (µM). Please view the correct S2 Table below.

## Supporting Information

S2 Table Composition of nutrient solutions. (XLSX)
