# Supplementary figures and images for: Phosphorus and Nitrogen Regulate Arbuscular Mycorrhizal Symbiosis in Petunia hybrida
Source: PLoS One. 2014 Mar 7;9(3):e90841. doi: 10.1371/journal.pone.0090841 (PMC3946601; doi:10.1371/journal.pone.0090841)

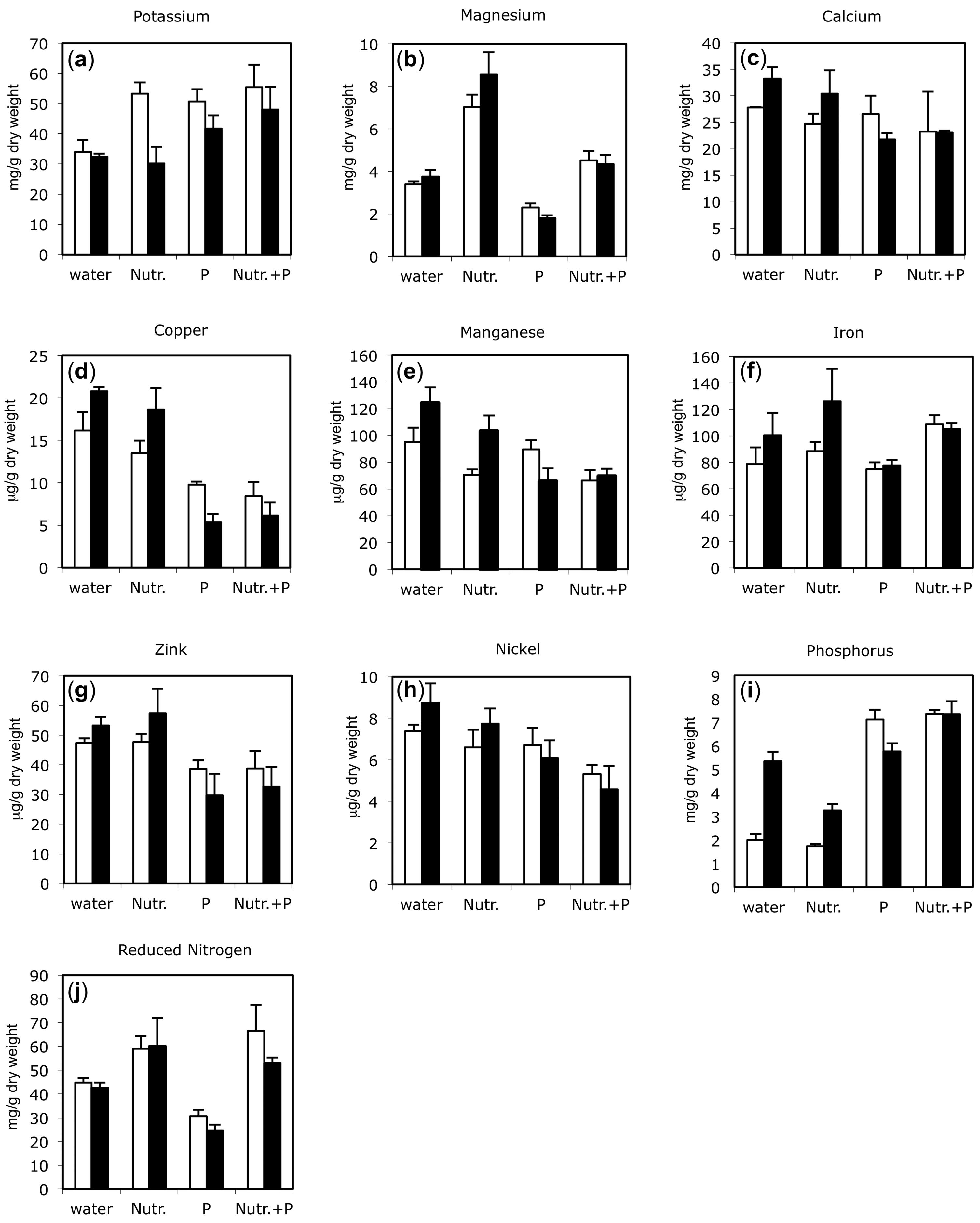

Supplement: Figure S1 — Nutrient content of mycorrhizal and non-mycorrhizal plants under different nutritional treatments. Nutrient levels were determined in the leaves of plants treated with only water, with basic nutrient solution (Nutr.), with 5 mM KH2PO4 (P), or with a combination of nutrient solution and 5 mM KH2PO4 (Nutr.+P). Plants were harvested 36 days after inoculation with R. irregularis (black columns) or mock inoculation (white columns). Columns represent the average of three biological replicates, error bars represent the standard deviations. (TIF) [file pone.0090841.s001.tif]
