# Supplementary material for: Phosphorus and Nitrogen Regulate Arbuscular Mycorrhizal Symbiosis in Petunia hybrida
Source: PLoS One. 2014 Mar 7;9(3):e90841. doi: 10.1371/journal.pone.0090841 (PMC3946601; doi:10.1371/journal.pone.0090841)
Supplement: File S1 — Predicted amino acid sequences of nitrate and nitrite transporters of Arabidopsis thaliana and Petunia hybrida used for phylogenetic analysis. (DOCX) [file pone.0090841.s002.docx]

Predicted amino acid sequences of nitrate and nitrite transporters of *Arabidopsis thaliana* and *Petunia hybrida* used for phylogenetic analysis (Nouri et al.; Figure 7)

>AtNRT1-1

MSLPETKSDDILLDAWDFQGRPADRSKTGGWASAAMILCIEAVERLTTLGIGVNLVTYLTGTMHLGNATAANTVTNFLGTSFMLCLLGGFIADTFLGRYLTIAIFAAIQATGVSILTLSTIIPGLRPPRCNPTTSSHCEQASGIQLTVLYLALYLTALGTGGVKASVSGFGSDQFDETEPKERSKMTYFFNRFFFCINVGSLLAVTVLVYVQDDVGRKWGYGICAFAIVLALSVFLAGTNRYRFKKLIGSPMTQVAAVIVAAWRNRKLELPADPSYLYDVDDIIAAEGSMKGKQKLPHTEQFRSLDKAAIRDQEAGVTSNVFNKWTLSTLTDVEEVKQIVRMLPIWATCILFWTVHAQLTTLSVAQSETLDRSIGSFEIPPASMAVFYVGGLLLTTAVYDRVAIRLCKKLFNYPHGLRPLQRIGLGLFFGSMAMAVAALVELKRLRTAHAHGPTVKTLPLGFYLLIPQYLIVGIGEALIYTGQLDFFLRECPKGMKGMSTGLLLSTLALGFFFSSVLVTIVEKFTGKAHPWIADDLNKGRLYNFYWLVAVLVALNFLIFLVFSKWYVYKEKRLAEVGIELDDEPSIPMGH

>AtNRT1-4

MESKGSWTVADAVDYKGRPADKSKTGGWITAALILGIEVVERLSTMGIAVNLVTYLMETMHLPSSTSANIVTDFMGTSFLLCLLGGFLADSFLGRFKTIGIFSTIQALGTGALAVATKLPELRPPTCHHGEACIPATAFQMTILYVSLYLIALGTGGLKSSISGFGSDQFDDKDPKEKAHMAFFFNRFFFFISMGTLLAVTVLVYMQDEVGRSWAYGICTVSMAIAIVIFLCGTKRYRYKKSQGSPVVQIFQVIAAAFRKRKMELPQSIVYLYEDNPEGIRIEHTDQFHLLDKAAIVAEGDFEQTLDGVAIPNPWKLSSVTKVEEVKMMVRLLPIWATTIIFWTTYAQMITFSVEQASTMRRNIGSFKIPAGSLTVFFVAAILITLAVYDRAIMPFWKKWKGKPGFSSLQRIAIGLVLSTAGMAAAALVEQKRLSVAKSSSQKTLPISVFLLVPQFFLVGAGEAFIYTGQLDFFITQSPKGMKTMSTGLFLTTLSLGFFVSSFLVSIVKRVTSTSTDVGWLADNINHGRLDYFYWLLVILSGINFVVYIICALWFKPTKGKDSVEKENGKGFSVEDC

>AtNRT1-2

MVHVSSSHGAKDGSEEAYDYRGNPPDKSKTGGWLGAGLILGSELSERICVMGISMNLVTYLVGDLHISSAKSATIVTNFMGTLNLLGLLGGFLADAKLGRYKMVAISASVTALGVLLLTVATTISSMRPPICDDFRRLHHQCIEANGHQLALLYVALYTIALGGGGIKSNVSGFGSDQFDTSDPKEEKQMIFFFNRFYFSISVGSLFAVIALVYVQDNVGRGWGYGISAATMVVAAIVLLCGTKRYRFKKPKGSPFTTIWRVGFLAWKKRKESYPAHPSLLNGYDNTTVPHTEMLKCLDKAAISKNESSPSSKDFEEKDPWIVSTVTQVEEVKLVMKLVPIWATNILFWTIYSQMTTFTVEQATFMDRKLGSFTVPAGSYSAFLILTILLFTSLNERVFVPLTRRLTKKPQGITSLQRIGVGLVFSMAAMAVAAVIENARREAAVNNDKKISAFWLVPQYFLVGAGEAFAYVGQLEFFIREAPERMKSMSTGLFLSTISMGFFVSSLLVSLVDRVTDKSWLRSNLNKARLNYFYWLLVVLGALNFLIFIVFAMKHQYKADVITVVVTDDDSVEKEVTKKESSEFELKDIP

>AtPTR1

MEEKDVYTQDGTVDIHKNPANKEKTGNWKACRFILGNECCERLAYYGMGTNLVNYLESRLNQGNATAANNVTNWSGTCYITPLIGAFIADAYLGRYWTIATFVFIYVSGMTLLTLSASVPGLKPGNCNADTCHPNSSQTAVFFVALYMIALGTGGIKPCVSSFGADQFDENDENEKIKKSSFFNWFYFSINVGALIAATVLVWIQMNVGWGWGFGVPTVAMVIAVCFFFFGSRFYRLQRPGGSPLTRIFQVIVAAFRKISVKVPEDKSLLFETADDESNIKGSRKLVHTDNLKFFDKAAVESQSDSIKDGEVNPWRLCSVTQVEELKSIITLLPVWATGIVFATVYSQMSTMFVLQGNTMDQHMGKNFEIPSASLSLFDTVSVLFWTPVYDQFIIPLARKFTRNERGFTQLQRMGIGLVVSIFAMITAGVLEVVRLDYVKTHNAYDQKQIHMSIFWQIPQYLLIGCAEVFTFIGQLEFFYDQAPDAMRSLCSALSLTTVALGNYLSTVLVTVVMKITKKNGKPGWIPDNLNRGHLDYFFYLLATLSFLNFLVYLWISKRYKYKKAVGRAH

>AtPTR5

MEDDKDIYTKDGTLDIHKKPANKNKTGTWKACRFILGTECCERLAYYGMSTNLINYLEKQMNMENVSASKSVSNWSGTCYATPLIGAFIADAYLGRYWTIASFVVIYIAGMTLLTISASVPGLTPTCSGETCHATAGQTAITFIALYLIALGTGGIKPCVSSFGADQFDDTDEKEKESKSSFFNWFYFVINVGAMIASSVLVWIQMNVGWGWGLGVPTVAMAIAVVFFFAGSNFYRLQKPGGSPLTRMLQVIVASCRKSKVKIPEDESLLYENQDAESSIIGSRKLEHTKILTFFDKAAVETESDNKGAAKSSSWKLCTVTQVEELKALIRLLPIWATGIVFASVYSQMGTVFVLQGNTLDQHMGPNFKIPSASLSLFDTLSVLFWAPVYDKLIVPFARKYTGHERGFTQLQRIGIGLVISIFSMVSAGILEVARLNYVQTHNLYNEETIPMTIFWQVPQYFLVGCAEVFTFIGQLEFFYDQAPDAMRSLCSALSLTAIAFGNYLSTFLVTLVTKVTRSGGRPGWIAKNLNNGHLDYFFWLLAGLSFLNFLVYLWIAKWYTYKKTTGHAL

>AtNRT2-1

MGDSTGEPGSSMHGVTGREQSFAFSVQSPIVHTDKTAKFDLPVDTEHKATVFKLFSFAKPHMRTFHLSWISFSTCFVSTFAAAPLVPIIRENLNLTKQDIGNAGVASVSGSIFSRLVMGAVCDLLGPRYGCAFLVMLSAPTVFSMSFVSDAAGFITVRFMIGFCLATFVSCQYWMSTMFNSQIIGLVNGTAAGWGNMGGGITQLLMPIVYEIIRRCGSTAFTAWRIAFFVPGWLHIIMGILVLNLGQDLPDGNRATLEKAGEVAKDKFGKILWYAVTNYRTWIFVLLYGYSMGVELSTDNVIAEYFFDRFHLKLHTAGLIAACFGMANFFARPAGGYASDFAAKYFGMRGRLWTLWIIQTAGGLFCVWLGRANTLVTAVVAMVLFSMGAQAACGATFAIVPFVSRRALGIISGLTGAGGNFGSGLTQLLFFSTSHFTTEQGLTWMGVMIVACTLPVTLVHFPQWGSMFLPPSTDPVKGTEAHYYGSEWNEQEKQKNMHQGSLRFAENAKSEGGRRVRSAATPPENTPNNV

>AtNRT2-6

MAHNHSNEDGSIGTSLHGVTAREQVFSFSVQEDVPSSQAVRTNDPTAKFALPVDSEHRAKVFKPLSFAKPHMRAFHLGWISFFTCFISTFAAAPLVPVIRDNLDLTKTDIGNAGVASVSGAIFSRLAMGAVCDLLGARYGTAFSLMLTAPAVFSMSFVADAGSYLAVRFMIGFCLATFVSCQYWTSVMFTGKIIGLVNGCAGGWGDMGGGVTQLLMPMVFHVIKLTGATPFTAWRFAFFIPGILQIVMGILVLTLGQDLPDGNLSTLQKSGQVSKDKFSKVFWFAVKNYRTWILFMLYGFSMGVELTINNVISGYFYDRFNLTLHTAGIIAASFGMANFFARPFGGYASDVAARLFGMRGRLWILWILQTVGALFCIWLGRASSLPIAILAMMLFSMGTQAACGALFGVAPFVSRRSLGLISGLTGAGGNFGSGVTQLLFFSSSRFSTAEGLSLMGVMAVVCSLPVAFIHFPQWGSMFLRPSQDGEKSKEEHYYGAEWTEEEKSLGLHEGSIKFAENSRSERGRKAMLADIPTPETGSPAHV

>AtNRT2-3

MTHNHSNEEGSIGTSLHGVTAREQVFSFSVDASSQTVQSDDPTAKFALPVDSEHRAKVFNPLSFAKPHMRAFHLGWLSFFTCFISTFAAAPLVPIIRDNLDLTKTDIGNAGVASVSGAIFSRLAMGAVCDLLGARYGTAFSLMLTAPTVFSMSFVGGPSGYLGVRFMIGFCLATFVSCQYWTSVMFNGKIIGLVNGCAGGWGDMGGGVTQLLMPMVFHVIKLAGATPFMAWRIAFFVPGFLQVVMGILVLSLGQDLPDGNLSTLQKSGQVSKDKFSKVFWFAVKNYRTWILFVLYGSSMGIELTINNVISGYFYDRFNLKLQTAGIVAASFGMANFIARPFGGYASDVAARVFGMRGRLWTLWIFQTVGALFCIWLGRASSLPIAILAMMLFSIGTQAACGALFGVAPFVSRRSLGLISGLTGAGGNFGSGLTQLLFFSSARFSTAEGLSLMGVMAVLCTLPVAFIHFPQWGSMFLRPSTDGERSQEEYYYGSEWTENEKQQGLHEGSIKFAENSRSERGRKVALANIPTPENGTPSHV

>AtNRT2-4

MADGFGEPGSSMHGVTGREQSYAFSVESPAVPSDSSAKFSLPVDTEHKAKVFKLLSFEAPHMRTFHLAWISFFTCFISTFAAAPLVPIIRDNLNLTRQDVGNAGVASVSGSIFSRLVMGAVCDLLGPRYGCAFLVMLSAPTVFSMSFVGGAGGYITVRFMIGFCLATFVSCQYWMSTMFNGQIIGLVNGTAAGWGNMGGGVTQLLMPMVYEIIRRLGSTSFTAWRMAFFVPGWMHIIMGILVLTLGQDLPDGNRSTLEKKGAVTKDKFSKVLWYAITNYRTWVFVLLYGYSMGVELTTDNVIAEYFFDRFHLKLHTAGIIAASFGMANFFARPIGGWASDIAARRFGMRGRLWTLWIIQTLGGFFCLWLGRATTLPTAVVFMILFSLGAQAACGATFAIIPFISRRSLGIISGLTGAGGNFGSGLTQLVFFSTSTFSTEQGLTWMGVMIMACTLPVTLVHFPQWGSMFLPSTEDEVKSTEEYYYMKEWTETEKRKGMHEGSLKFAVNSRSERGRRVASAPSPPPEHV

>AtNRT2-7

MEPSQRNTKPPSFSDSTIPVDSDGRATVFRPFSLSSPHSRAFHLAWLSLFSCFFSTFSIPPLVPVISSDLNLSASTVSAAGIASFAGSIFSRLAMGPLCDLIGPRTSSAILSFLTAPVILSASLVSSPTSFILVRFFVGFSLANFVANQYWMSSMFSGNVIGLANGVSAGWANVGAGISQLLMPLIYSTIAEFLPRAVAWRVSFVFPAIFQVTTAVLVLLYGQDTPHGNRKNSNQNKLTIPEEEEVLVVEEDERSSFVEILIGGLGNYRAWILALLYGYSYGVELTTDNVIAGYFYERFGVNLEAAGTIAASFGISNIASRPAGGMISDALGKRFGMRGRLWGLWIVQSVAGLLCVLLGRVNSLWGSILVMWVFSVFVQAASGLVFGVVPFVSTRSLGVVAGITGSGGTVGAVVTQFLLFSGDDVRKQRSISLMGLMTFVFALSVTSIYFPQWGGMCCGPSSSSEEEDISRGLLVEDEDEEGKVVSGSLRPVC

>AtNRT2-5

MEVEGKGGEAGTTTTTAPRRFALPVDAENKATTFRLFSVAKPHMRAFHLSWFQFFCCFVSTFAAPPLLPVIRENLNLTATDIGNAGIASVSGAVFARIVMGTACDLFGPRLASAALTLSTAPAVYFTAGIKSPIGFIMVRFFAGFSLATFVSTQFWMSSMFSGPVVGSANGIAAGWGNLGGGATQLIMPIVFSLIRNMGATKFTAWRIAFFIPGLFQTLSAFAVLLFGQDLPDGDYWAMHKSGEREKDDVGKVISNGIKNYRGWITALAYGYCFGVELTIDNIIAEYFFDRFHLKLQTAGIIAASFGLANFFARPGGGIFSDFMSRRFGMRGRLWAWWIVQTSGGVLCACLGQISSLTVSIIVMLVFSVFVQAACGLTFGVVPFISRRSLGVVSGMTGAGGNVGAVLTQLIFFKGSTYTRETGITLMGVMSIACSLPICLIYFPQWGGMFCGPSSKKVTEEDYYLAEWNDEEKEKNLHIGSQKFAETSISERGRATTTHPQT

>AtNRT2-2

MGSTDEPGSSMHGVTGREQSYAFSVDGSEPTNTKKKYNLPVDAEDKATVFKLFSFAKPHMRTFHLSWISFSTCFVSTFAAAPLIPIIRENLNLTKHDIGNAGVASVSGSIFSRLVMGAVCDLLGPRYGCAFLVMLSAPTVFSMSFVSDAAGFITVRFMIGFCLATFVSCQYWMSTMFNSQIIGLVNGTAAGWGNMGGGITQLLMPIVYEIIRRCGSTAFTAWRIAFFVPGWLHIIMGILVLTLGQDLPGGNRAAMEKAGEVAKDKFGKILWYAVTNYRTWIFVLLYGYSMGVELSTDNVIAEYFFDRFHLKLHTAGIIAACFGMANFFARPAGGWASDIAAKRFGMRGRLWTLWIIQTSGGLFCVWLGRANTLVTAVVSMVLFSLGAQAACGATFAIVPFVSRRALGIISGLTGAGGNFGSGLTQLVFFSTSRFTTEEGLTWMGVMIVACTLPVTLIHFPQWGSMFFPPSNDSVDATEHYYVGEYSKEEQQIGMHLKSKLFADGAKTEGGSSVHKGNATNNA

>AtNitr1

MEEQSKNKISEEEKQLHGRPNRPKGGLITMPFIFANEICEKLAVVGFHANMISYLTTQLHLPLTKAANTLTNFAGTSSLTPLLGAFIADSFAGRFWTITFASIIYQIGMTLLTISAIIPTLRPPPCKGEEVCVVADTAQLSILYVALLLGALGSGGIRPCVVAFGADQFDESDPNQTTKTWNYFNWYYFCMGAAVLLAVTVLVWIQDNVGWGLGLGIPTVAMFLSVIAFVGGFQLYRHLVPAGSPFTRLIQVGVAAFRKRKLRMVSDPSLLYFNDEIDAPISLGGKLTHTKHMSFLDKAAIVTEEDNLKPGQIPNHWRLSTVHRVEELKSVIRMGPIGASGILLITAYAQQGTFSLQQAKTMNRHLTNSFQIPAGSMSVFTTVAMLTTIIFYDRVFVKVARKFTGLERGITFLHRMGIGFVISIIATLVAGFVEVKRKSVAIEHGLLDKPHTIVPISFLWLIPQYGLHGVAEAFMSIGHLEFFYDQAPESMRSTATALFWMAISIGNYVSTLLVTLVHKFSAKPDGSNWLPDNNLNRGRLEYFYWLITVLQAVNLVYYLWCAKIYTYKPVQVHHSKEDSSPVKEELQLSNRSLVDE

>PhNRT2_cn8666

MGDIEGEPGSSMHGVTGREPVLAFSVASPMVPTDTTAKFSVPVDTEHKAKVFKFYSFSKPHGLTFQLSWISFFTCFVSTFAAAPLVPIIRDNLNLTKMDVGNAGVASVSGSILSRLVMGAVCDLLGPRYGCAFLIMLSAPTVFCMSFVSSAGGYVAVRFMIGFSLATFVSCQYWMSTMFNSQIIGLVNGTAAGWGNMGGGATQLLMPLLYDIIRRTGSTPFTAWRIAFFIPGWLHVVMGILVLTLGQDLPDGNRGSLQKTGTVAKDKFGNILWYAATNYRTWIFVLLYGYSMGVELSTDNVIAEYFFDRFDLKLHTAGIIAATFGMANLLARPFGGFSSDYAAKKFGMRGRLWVLWILQTLGGVFCVLLGRSNSLPIAVTFMILFSIGAQAACGATFGIIPFISRRSLGIISGMTGAGGNFGSGLTQLLFFTSSKYSTATGLTYMGIMIIGCTLPVTLCHFPQWGSMFLPPSKDPVKGTEEHYYTSEYTEAEKQKGMHQGSLKFAENCRSERGKRVGSAPTPPNLTPNRV

>PhNRT2b_cn7864

MGDIEGEPGSSMHGVTGREPVLAFSVASPMVPTDTTAKFSVPVDTEHKAKVFKFYSFSKPHGLTFQLSWISFFTCFVSTFAAAPLVPIIRDNLNLTKMDVGNAGVASVSGSILSRLVMGAVCDLLGPRYGCAFLIMLSAPTVFCMSFVSSAGGYVAVRFMIGFSLATFVSCQYWMSTMFNSQIIGLVNGTAAGWGNMGGGATQLLMPLLYDIIRRTGSTPFTAWRIAFFIPGWLHVVMGILVLTLGQDLPDGNRGSLQQTGTVAKDKFGNILWYAATNYRTWIFVLLYGYSMGVELSTDNVIAEYFFDRFDLKLHTAGIIAATFGMANLLARPFGGFSSDYAAKKFGMRGRLWVLWILQTLGGVFCVLLGRSNSLPIAITFMILFSIGAQAACGATFGIIPFISRRSLGIISGMTGAGGNFGSGLTQLLFFTSSKYSTATGLTYMGIMIIGCTLPVTLCHFPQWGSMFLPPSKDPVKGTEEHYYTSEYTEDERQKGMHQGSLKFAENCRSERGKRVASAPTPPNLTPNRV

>PhNRT1_cn5272

MEESKKSTTAWFACCTKCLPFSSSKSSSLSFPSPEKSISYDEEKQGCSDATSRRKPGGWRAMPYVLGNETFERLASIGLLANFMQFLLTQFHMDQVSASNVLNIWSGFTNFIPLLGAYLSDAYVGRFWTIAFASVFETMGMLTLTMIPWLPKLHPPPCKVGQKCQGPNKSQMGFLALGLGFLSIGSGGIRPCSIPFGVDQFDSTTNEGRKGIASFFNWYYTSFTVVLIIALTLVVCIQDSVSWVIGVGIPTVLMFLSLILFFIGTRVYVHVKPEGSIFSSIVQVFVVTYKKRKLKLSDEHGSNDVVLYDPPPQIGTIVKKLPLTNKYRSLNKAAIVMEGEVNTDGTRSNKWRLCSIQQIEEVKCLLQIIPVWASGIICFTAMAQQGTFTMSQALKMDRHLGPNFQIPAGSLSVISMITVGVWLPIYDRLIVPSIRKRTGNDGGITLLQRVGIGMVFSILSMVAAGLIEKVRRDSAIMHNSPDGIAPITVMWLAPQLILMGFAEAFNILGPIEFFHKEFPEHMSSLANSLFSVTVAGASYLSSLLVNILHKTTGGHGHPDWLTKDINEGRIENFYYLIAGLGVLNLFYFIYVSRQYQYKTRLVVDDGIKPFNSDVALHDMKY

>PhNRT1b_SGN-U210769

MEVNKEEVSAKNEPKYPGIKAMPFIIGNETFEKLGTIGTSSNLLVYLTTVFNMKSITATNLINVFNGTCNFGTLLGAFLTDTYLGRYKTLGIASISSFLGMLFLTLTAAISKLHPPHCGTEKDSICLEPTTGQLAFLLCSFGFLVVGASGIRPCNLAFGADQFNPNTESGRRGNNSFFNWYYLTFTFAMMVSLTVIVYIQSSVNWALGLAIPTFLMFLSCVFFFIGTKIYVMILPEGSPLTSMVQVLVAAIKKRRLKLPEQPQDTLFNHVSTNTVNSELPYTDQFRFLNKACIITPEDRIKEDGSAANPWRLCRIQQVEEVKCVVRVFPIWIAGLVYYIVLVQMQTYVVFQALQSDRRLVKSSNFQVPAATYAVFSMLSMTIWILIYDRIVVPFLRKITKKEAGITMLQRMGIGLFISIFTVIISAVVETRRRNMALSHPTLGNGSRKGEVSAMSANWLIPQLALAGISEAFTVIAEVEFFYKQFPENMRSFAGSFLFCGFALASYASTMLISIVHRTTRISDTENWLAEDLNKGRLDYFYYLVAALEVFNLVYFLICAKWYKYKGTQNHNLEVSMEKLEPTKPLV

>PhNitr1_CL1918_cn5943

MKGGMITMPFIFANEICEKLAVVGFGANMIIYLTNELHLPLTKAANTLTNFGGTASLTPLLGAFLADTFAGRFWTITVASIIYQIGMIILTLSAILPQLRPPSCKDDEICKEANSGQLAILYVSLLLTAFGSGGIRPCVVAFGADQFDETDPKQKTQTWKFFNWYYFCMGVSMLVAVTVVVYIQDNIGWGVGFGVPTIAMLISIIVFIFGYPLYRNLDPAGSPFTRLLQVCVAAYKKRKLPMVSDPSYLYQNGELDAAISTAGKLVHTKQMKFLDRAAIVTEEDSPKSPNLWRLNTVHRIEELKSVIRMGPIWASGIILITAYAQQHTFSVQQAKTMDRHLTNSFQIPAASMTVFTLIAMLCTIAFYDRVFVPIARRFTGLERGISFLSRMAIGFFISVLATLVAGFIEVKRKEAALAHGLIDKSKAIVPISVFWLVPQYCLHGIAEAFMSIGHLEFFYDQAPESMRSTATALFWTSISAGNYLSTLLVSLVHKFTSGPGGSNWLPDDNLNKGKLEYFYWLITILQVVNLIYYLFCAKFYTFKPIQVQNTEDLDTKKDVELVNNV
